# Intratumoral Cell Heterogeneity in Patient-Derived Glioblastoma Cell Lines Revealed by Single-Cell RNA-Sequencing

**DOI:** 10.3390/ijms25158472

**Published:** 2024-08-02

**Authors:** Mikhail Arbatskiy, Dmitriy Balandin, Alexey Churov, Vyacheslav Varachev, Eugenia Nikolaeva, Alexei Mitrofanov, Ali Bekyashev, Olga Tkacheva, Olga Susova, Tatiana Nasedkina

**Affiliations:** 1 Russian Clinical Research Center of Gerontology, Pirogov Russian National Research Medical University of the Ministry of Healthcare of the Russian Federation, 129226 Moscow, Russiaachurou@yandex.ru (A.C.); tkacheva@rgnkc.ru (O.T.); 2 Engelhardt Institute of Molecular Biology, The Russian Academy of Sciences, 119991 Moscow, Russia; varachevviacheslav95@mail.ru (V.V.); tanased06@rambler.ru (T.N.); 3 N.N. Blokhin National Medical Research Center of Oncology, Ministry of Health of the Russian Federation, 115522 Moscow, Russia; jane.niko.biochem@gmail.com (E.N.); mitrofanov-aa@list.ru (A.M.); abekyashev@gmail.com (A.B.); o.susova@ronc.ru (O.S.)

**Keywords:** glioblastoma, intratumor heterogeneity, patient-derived cell lines, single-cell RNA sequencing

## Abstract

Glioblastoma cell lines derived from different patients are widely used in tumor biology research and drug screening. A key feature of glioblastoma is the high level of inter- and intratumor heterogeneity that accounts for treatment resistance. Our aim was to investigate whether intratumor heterogeneity is maintained in cell models. Single-cell RNA sequencing was used to investigate the cellular composition of a tumor sample and six patient-derived glioblastoma cell lines. Three cell lines preserved the mutational profile of the original tumor, whereas three others differed from their precursors. Copy-number variation analysis showed significantly rearranged genomes in all the cell lines and in the tumor sample. The tumor had the most complex cell composition, including cancer cells and microenvironmental cells. Cell lines with a conserved genome had less diverse cellularity, and during cultivation, a relative increase in the stem-cell-derived progenitors was noticed. Cell lines with genomes different from those of the primary tumors mainly contained neural progenitor cells and microenvironmental cells. The establishment of cell lines without the driver mutations that are intrinsic to the original tumors may be related to the selection of clones or cell populations during cultivation. Thus, patient-derived glioblastoma cell lines differ substantially in their cellular profile, which should be taken into account in translational studies.

## 1. Introduction

Glioblastoma is the most common and aggressive type of adult-type diffuse glioma, with a median survival time after surgical treatment followed by chemoradiation therapy of about 15 months and a 5-year survival rate of less than 5% [1,2]. According to the 2021 WHO Classification of Tumors of the Central Nervous System (CNS), glioblastoma isocitrate dehydrogenase (IDH)-wildtype represents a definite subtype, which is characterized by specific molecular and histopathological features [3]. A hallmark of glioblastoma is the high degree of genomic, epigenomic, metabolic, and microenvironmental variability, both intertumor heterogeneity between affected patients and intratumor heterogeneity within individual tumors [4,5,6].

The term “glioblastoma multiforme” was suggested by pathologists who were the first to note the highly variable morphology of the tumor cells. In addition to cellular morphology, heterogeneity can present as diversity in mutations and structural variants, differences in chromatin landscapes, and transcriptional regulation. An important contribution to understanding the molecular landscape of glioblastoma and inter-patient heterogeneity has been made by the Cancer Genome Atlas (TCGA) project, which has performed comprehensive genomic and transcriptomic analysis on hundreds of tumors [7]. Based on transcriptome analysis, a division into four tumor subtypes was proposed: proneural (PN), classical (CL), mesenchymal (MES), and neural (NL) [8]. Other studies using bulk RNA sequencing have also appealed to this classification [9]. 

Initially, intratumor heterogeneity was investigated when biopsy specimens were taken from spatially separated areas of a single tumor [4]. The transition to a new level in studies of intratumor heterogeneity, including highly heterogeneous glioblastoma, became possible with the development of single-cell approaches with dramatically increased resolution [10]. Single-cell RNA sequencing (scRNA-seq) has become a key method for comprehensively analyzing cellular states in tissues, both in normal conditions and during pathological processes. It was shown by scRNA-seq that a single glioblastoma tumor consisted of a heterogeneous mixture of cells representing a wide variety of subgroups [11]. Malignant glioblastoma cells from different patients have been found to exist in four reproducible cell states, namely neural progenitor like (NPC-like), oligodendrocyte progenitor like (OPC-like), astrocyte like (AC-like), and mesenchymal like (MES-like) [12]. Moreover, intratumor heterogeneity includes non-malignant cells in the tumor microenvironment, such as neuronal, glial, and immune populations, as well as endothelial cells, which can functionally interact with cancer cells and promote tumor development [13].

Understanding intratumor heterogeneity in glioblastoma is essential for unraveling the molecular mechanisms driving tumor development and progression, as well as for devising effective treatment strategies. Intratumor heterogeneity is a significant challenge in the treatment of glioblastoma, as it serves as a natural pool for the growth of treatment-resistant clones, leading to poor therapeutic efficacy. While progress has been made in identifying the link between mutational drivers and deterministic cell fate, the development and validation of robust experimental models are still critical.

Various glioblastoma models are widely used to study tumor biology, screen for antitumor-drug efficacy, and investigate the mechanisms of therapy-resistance development: (1) primary cell cultures derived directly from patient tumor samples (2D cultures, i.e., growing on a flat surface) (patient-specific cultures) [14,15]; (2) 3D primary cell cultures (neurospheres, patient-derived explants PDE, organoids) [16]; (3) subcutaneous and orthotopic glioblastoma xenografts in mouse models [17].

Patient-derived cell lines are the most widely used models of glioblastoma due to their relative ease of establishment and the ability to be frozen and stored for long periods while retaining the basic characteristics of the original culture. An important aspect of using patient-derived cell lines in the study of glioblastoma biology and drug screening is understanding the extent to which in vitro cultivation can preserve (1) the genetic characteristics of primary tumors and (2) the diversity of cell types based on transcriptional signatures as a reflection of intratumor heterogeneity.

This work aimed to investigate the intratumor heterogeneity of primary cell cultures derived from the glioblastoma tumors of different patients compared to a glioblastoma-operating tumor sample. Single-cell RNA sequencing was used to analyze one glioblastoma tumor (Gbt) sample and six patient-derived cell lines (Gbl) from different patients. Three of them had the same driver mutations as the original tumor, while the other three showed different genetic aberrations. In addition, we compared the composition of cell populations in patient-derived cell lines with cell-culture passage to trace the possible evolution of tumor cells during cultivation. 

Our data provide evidence that heterogeneity in glioblastoma due to intrinsic cancer cell plasticity may be retained in patient-derived cell lines, which has important implications for the design of treatment strategies. Thus, this single-cell study will contribute novel insights into the cellular landscape for deciphering intratumor heterogeneity in patient-derived cell lines compared to tumor, with valuable significance for model selecting in glioblastoma research.

## 2. Results

### 2.1. Characteristics of Samples Involved in scRNA-Seq Study

To investigate the cellular heterogeneity and molecular signatures in patient-derived glioblastoma cell lines compared to a tumor, we generate single-cell RNA profiles from one glioblastoma tumor sample (Gbt) and six primary glioblastoma cell lines (Gbl) at different passages by 10× single-cell RNA sequencing. All samples belong to IDH-wildtype diffuse gliomas. Driver mutations in genes related to the development of glioblastoma were analyzed using multi-gene panel testing of 812 cancer-associated genes in bulk DNA samples. The clinical and genetic characteristics of the samples are given in Table 1. 

It was found that the three cell lines, Gbl27, Gbl13, and Gbl17, shared common driver mutations with the original tumor. Most notably, a mutation in the *PTEN* gene and a loss of heterozygosity (LOH) in the Gbl17, Gbl27, and Gbl13 cell lines were observed. However, the mutational profiles of Gbl6, Gbl24, and Gbl28 did not coincide with their tumor precursors, which might result from tumor subclonality or the selection of clones without tumor-parental mutations during cultivation. Also, when using surgical material to obtain cell lines, we cannot exclude the presence of a sufficient number of cells from the peritumoral zone, which could preferentially grow in some cases. All cell-line samples were also compared with their matched peripheral blood samples, and the presence of corresponding germline mutation sets confirmed their affiliations to particular patients.

### 2.2. Copy-Number Variations (CNVs) in Gbl and Gbt Samples Identified by scRNA-Seq

**Figure 1:**
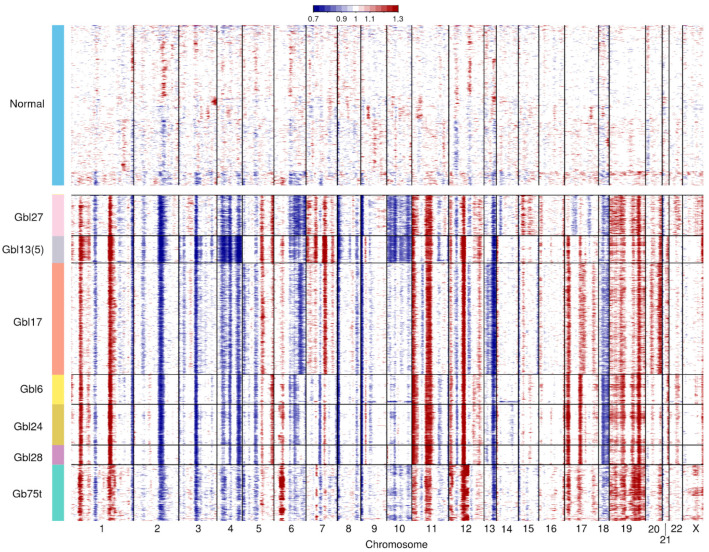
Copy-number aberrations detected in patient-derived glioblastoma cell lines and the tumor sample compared with normal brain genomic profile (at the top). Normal brain tissue data were obtained from GSE157827 (GSM4775574). The heatmaps show gains (red) and losses (blue) (Distribution of modified expression).

To confirm the tumor character of the cell lines on a genomic level, we used scRNA-seq data to analyze large-scale CNVs, including chromosomal rearrangements or full-chromosome events. This type of aberrations can be reliably detected on the basis of the average up- or down-regulation of large sets of genes within each chromosomal region. The results of the CNV analysis are in Figure 1. 

All Gbl samples and the tumor sample Gb75t had a highly rearranged genome compared to normal brain tissue. The rearrangements were more pronounced in Gbl27, Gbl13, and Gbl17, having conserved genome profiles from the original tumor tissue. In Gbl27 and Gbl13, the loss of chromosome 10 was observed, which was correlated with the LOH of the *PTEN* gene. Practically in all samples, the mostly noticed chromosomal rearrangements were gains in chromosomes 1, 11, 17, and 19; and losses in chromosomes 2, 4, and 13. Numerous copy-number aberrations were found in Gbl6, Gbl24, and Gbl28, as well as in tumor sample Gb75t, allowing us to consider these cell lines also to be of tumor origin. 

### 2.3. Tumor Phenotype of Gbt and Gbl Samples Confirmed by scRNA-Seq Analysis

**Figure 2:**
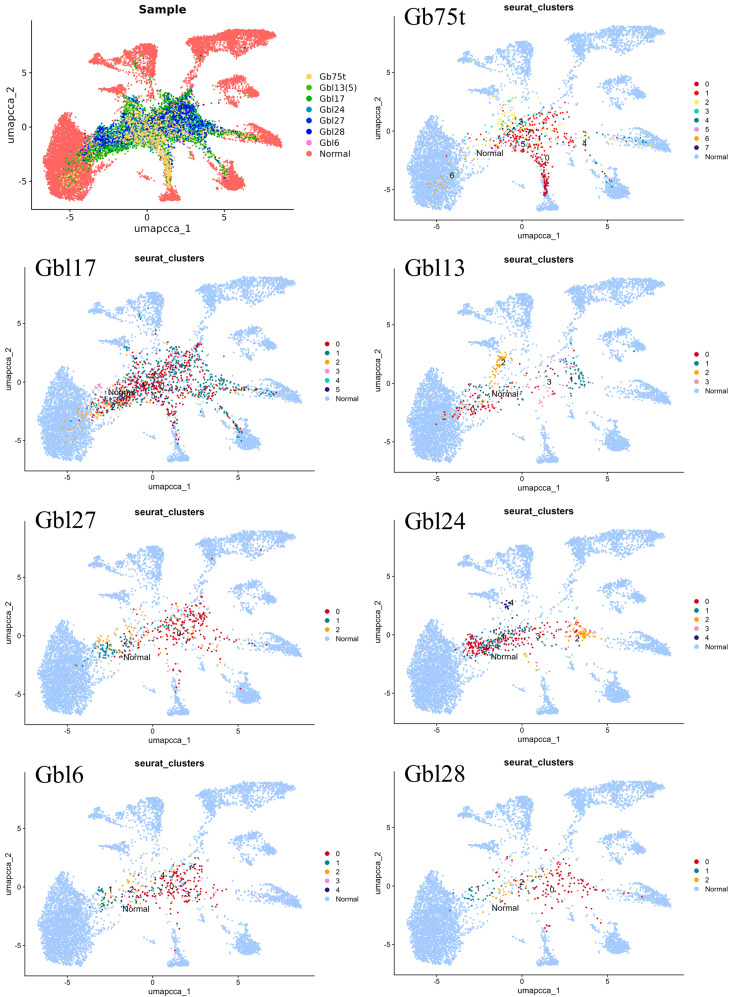
Integration of Gbt and Gbl samples with a sample of normal brain tissue. The uniform manifold approximation and projection (UMAP) plot of the tumor (Gb75t) and patient-derived cell line (Gbl) cell types among normal human brain cells.

Integrated transcriptome profiles of the Gbt and Gbl samples obtained from scRNA-seq data were compared with the scRNA-seq transcriptome profile of normal brain tissue (open data from scRNA-seq of brain tissue were used GSE157827 (GSM4775574) (Figure 2).

The UMAP dimensional reduction algorithm revealed clear structural separations based on molecular heterogeneity between the tumor and normal brain cells. We have found eight main segregated cell clusters in the normal brain, while none of the glioblastoma samples overlapped with it. Nevertheless, several inclusions of the Gb75t, Gbl13, and Gbl17 samples in the massif of normal tissue cells were observed, indicating the presence of some amount of non-malignant cells. In addition, bridges between the bulk of tumor cells and normal tissue were identified, which can be interpreted as an intermediate state between the normal and tumor phenotypes, but these cells were not abundant. A more detailed description regarding whether these cells belong to specific cell populations is given when analyzing the cell-clustering results for each sample. 

### 2.4. Cell Clustering of Glioblastoma Intraoperative Sample GB75t

The Gb75t specimen was obtained from the patient with glioblastoma IDH-wildtype. After scRNA-seq and gene-expression normalization, 1192 cells were retained for further analysis and cell clustering (Table S2).

**Figure 3:**
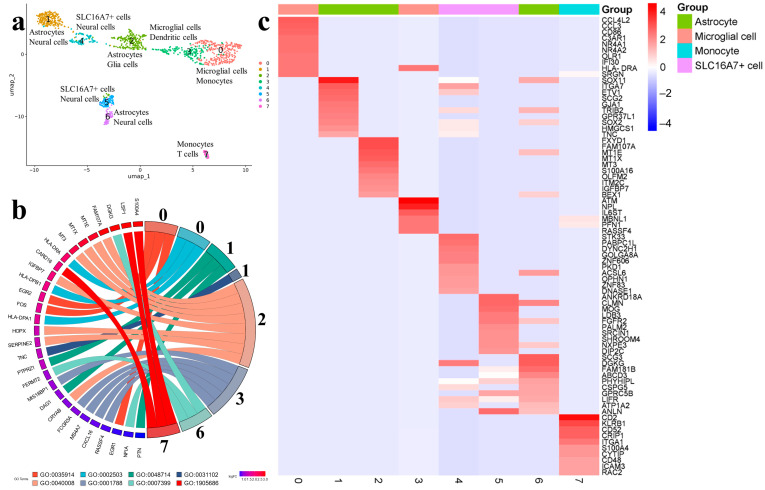
(**a**) UMAP plot of the scRNA-seq clustering from the intraoperative Gb75t sample from patient with glioblastoma IDH-wildtype. A total of eight clusters were found in this sample. Each cluster is colored differently and depicted by a number. Cell clusters were identified using two databases: CellMarker (upper line) and SingleCellBase (lower line); (**b**) GO Chord plot 0 cluster: GO:0035914 Skeletal muscle cell differentiation, GO:0002503 peptide antigen assembly with MHC class II protein complex, 1 cluster: GO:0048714 positive regulation of oligodendrocyte differentiation, GO:0031102 neuron projection regeneration, 2 cluster: GO:0040008 regulation of growth, 3 cluster: GO:0001788 antibody-dependent cellular cytotoxicity, 6 cluster: GO:0007399 nervous system development, 7 cluster: GO:1905686 positive regulation of plasma membrane repair (the full list of processes is presented in Table S3); (**c**) heatmap showing expression of the top genes associated with each cellular cluster (the full list of differentially expressed genes is presented in Supplementary Materials: Gb75t_heatmap.pdf).

Based on cell-specific markers and significantly enriched genes, the cells were categorized into eight clusters (Figure 3a). For each cluster, the identification of cell type using two databases (CellMarker and SingleCellBase), functional analysis, and the cell marker lists were received (Figure 3b,c). Further on, we prioritized the data generated by CellMarker and used the information from SingleCellBase as a supplementary (Supplementary Materials: Gb75t_info.xlsx). 

In the Gb75t, CellMarker identified three astrocyte clusters. Astrocytes 1 were enriched by 39 highly expressed markers, associated with oligodendrocyte differentiation (PTN, PTPRZ1, DAG1 GPR37L1), and neuron regeneration (SOX2, SOX11, TNC, PTN, DAG1) (Figure 3b,c). The markers FERMT2 and NFIA were common between two databases (Table S3). In astrocytes 2, 35 relevant markers were found that were involved in cell growth regulation (MT3, IGFBP7, MT1E, MT1X, SERPINE2, FAM107A, CRYAB, and HOPX). Astrocytes 6 represented a minor cluster with a high expression of 12 markers, and no specific processes were identified. Three common markers were found between two databases (DGKG, FERMT2, and NFIA). The expression pattern of astrocytes 6 was largely consistent with that of the normal brain tissue cell population, indicating that this cluster was presented mainly by non-malignant astrocytes (Figure 2, Gb75t). 

Microglial cells formed two clusters. Cell markers in microglial cells 0 (CCL4L2, CD86, EGR1, EGR2, FOS, NR4A1, NR4A2, GRN, HLA-DRA, IFI30, OLR1, and SRGN) were found to be involved in the regulation of interleukin-3 production, endothelial cell chemotaxis, and leukotriene production. In microglial cells 3 (or dendritic cells according to SingleCellBase), 140 markers were present, which were mainly associated with antibody-dependent cellular cytotoxicity. Five markers were common between two databases: CXCL16, FCGR3A, MIS18BP1, MS4A7, and RASSF4.

SLC16A7+ cells were categorized into minor clusters 4 and 5; each cluster included 23 markers unrelated to known biological processes. In the integration map (Figure 2, Gb75t), cells from cluster 4 had a diffuse distribution between areas of normal and tumor expression, likely indicating the heterogeneity of this cell population. Another minor cluster was presented by monocytes 7, expressing 68 markers mainly associated with the regulation of plasma membrane repair. Three common markers were found between two databases: CARD16, LSP1, and S100A4.

The trajectory tracing method was used to analyze the phylogenetic relationships between individual cell populations (Figure S1). Astrocytes 2 could be considered as the starting point, giving rise to the three directions: one direction was SLC16A7+ cells 4 leading to astrocytes 1, the second direction was SLC16A7+ cells 5 leading to astrocytes 6, and the third direction led to microglial cells 3. After bifurcation from microglial cells 3, the trajectory went on to microglial cells 0 and, further, to monocytes 7.

### 2.5. Cell Clustering of Gbl That Harbor the Same Mutations as Their Originating Tumor

Three cell lines, Gbl17, Gbl13, and Gbl 27, had mutational profiles very similar to the original tissue. The cell-culture samples were taken in the study at different passages: Gbl17 was on its third passage, Gbl13 on the fifth passage, and Gbl27 on the eleventh passage, relatively. Further, a description of the cellular composition and biological processes occurring in these samples is provided. 

After passing quality control and filtering criteria, 3826 individual cells per three samples were analyzed (2384 for Gbl17, 581 for Gbl13, and 861 for Gbl27) and clustered on the basis of the gene-expression profiles. 

**Figure 4:**
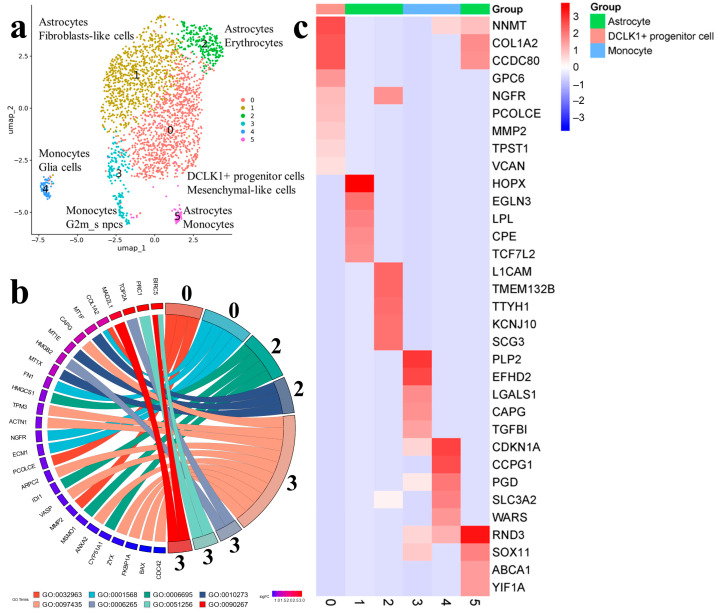
(**a**) Cell clusters identified in the Gbl17 (3 passage) using CellMarker (upper line) and SingleCellBase (lower line); G2m_s npcs (neural progenitor cells); (**b**) GO Chord plot 0 cluster: GO:0032963 collagen metabolic process, GO:0001568 blood vessel development, 2 cluster: GO:0006695 cholesterol biosynthetic process, GO:0010273 detoxification of copper ion, 3 cluster: GO:0097435 supramolecular fiber organization, GO:0006265 DNA topological change, GO:0051256 mitotic spindle midzone assembly, GO:0090267 positive regulation of mitotic cell cycle spindle assembly checkpoint (the full list of processes is presented in Table S4); (**c**) Heatmap showing expression of the top genes associated with each cellular cluster (the full list of differentially expressed genes is presented in Supplementary Materials: Gbl17_heatmap.pdf).

The results of cell clustering for the Gbl17 sample are in Figure 4a. 

According to the CellMarker database, cluster 0 is presented by DCLK1 + progenitor cells with high expression levels of COL1A2, MMP2, and PCOLCE, which participate in the metabolism of collagen. VCAN plays a role in intercellular signaling and in connecting cells with the extracellular matrix, and nerve growth factor receptor NGFR is important for the differentiation and survival of specific neuronal populations during development. (Figure 4c). SingleCellBase classified the cells as mesenchymal like, with the expression of genes participating in blood-vessel development COL1A2, FN1, NGFR, and ECM1 (Figure 4b). The cell markers COL1A2 and NGFR were presented in both databases (Table S4). 

Astrocytes were categorized into three major clusters. Astrocytes 1 expressed HOPX, CPE, EGLN3, LPL, and TCF7L2. Astrocytes 2 were specified by markers L1CAM (neural cell adhesion molecule), KCNJ10 (inwardly rectifying potassium channel), TTYH1 (probable chloride channel) and were also characterized by markers form the cholesterol biosynthetic process (CYP51A1, MSMO1, HMGCS1, and IDI1) (Figure 4b,c). Astrocytes 5 (or monocytes by SingleCellBase) formed a minor cluster. 

The other two minor clusters were presented by a noticeably smaller number of cells (n = 287) and were mostly identified as monocytes. Monocytes 3 (or G2/M cells) were enriched by cell markers of the mitotic cycle (HMGB2, TOP2A, BIRC5), cell differentiation and apoptosis (PLP2, EFHD2, and LGALS1), and macrophage function (CAPG). Monocytes 4 were characterized by cell markers of cellular energy processes (PGD, SLC3A2, HEBP2, NDUFS6, and COX5A) and cell proliferation (CCPG1, CDKN1A) (Supplementary Materials: Gbl17_info). A small amount of non-malignant cells were presented in astrocytes 1 and astrocytes 2 (Figure 2, Gbl17).

Single-cell trajectory analysis indicated the DCLK1+ progenitor cells 0 as the starting point giving rise to astrocytes 1, 2, and 5. The expression patterns of astrocytes 1 shifted to monocytes 4, while a small population of astrocytes 5 transited to actively proliferating monocytes 3 (Figure S2).

**Figure 5:**
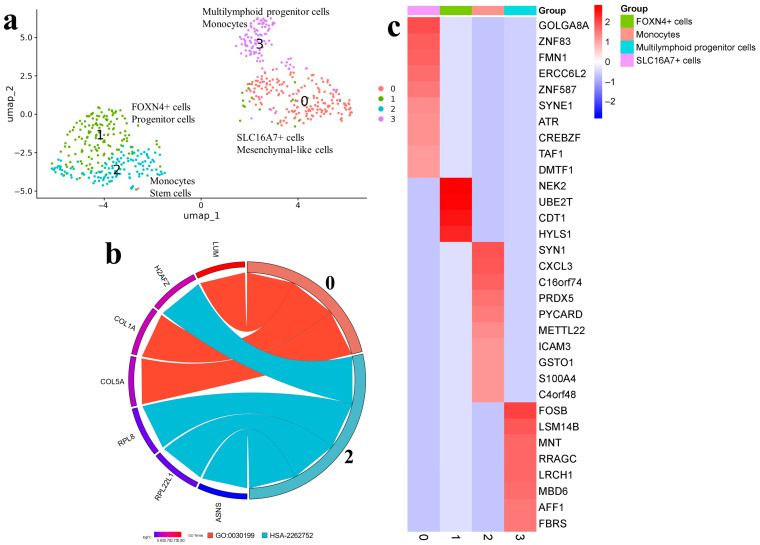
(**a**) Gbl13 (5 passage). Cell clusters identified in cell-culture sample using two databases: cell type assigned by CellMarker (upper line) and by SingleCellBase (lower line); (**b**) GO chord plot 0 cluster: GO:0030199 collagen fibril organization, 2 cluster: HSA-2262752 cellular responses to stress (the full list of processes is presented in Table S4); (**c**) Heatmap showing expression of the top genes associated with each cellular cluster (the full list of differentially expressed genes is presented in Supplementary Materials: Gbl13_heatmap.pdf).

The results of cell clustering for the Gbl13 sample are presented in Figure 5a. 

Cluster 0 was presented by SLC16A7+ cells (or mesenchymal-like cells) expressing SLC16A7, which is responsible for the transcription of the major pyruvate transporter MCT2. The differentially expressed markers were GOLGA8A, ZNF83 (DNA-binding transcription factor), FMN1, SYNE1, CREBZF (transcription factor) (Figure 5c). The markers COL1A, COL5A, and LUM were associated with collagen fibril organization (Figure 5b). In cluster 1, FOXN4+ cells (or progenitor cells) were categorized. Two markers, UBE2T and CDT1, were consistent across both databases. Monocytes 2 expressed RPL22L1, RPL8, ICAM3, and PRDX5, which are related to cellular responses to oxidative stress (Table S4). Cluster 3, according to the CellMarker, consisted of multilymphoid progenitor cells (Figure 5b,c; Supplementary Materials: Gbl13_info.xlsx).

Due to the analysis of trajectories, multilymphoid progenitor cells 3 were considered as the starting point, transited to SLC16A7+ cells 0 and FOXN4+ cells 1, and then to monocytes 2 (Figure S3).

**Figure 6:**
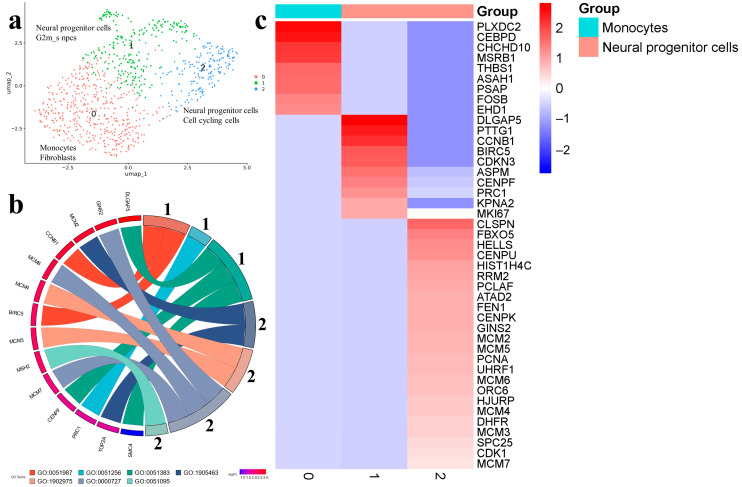
(**a**) Gbl27 (11 passage). Cell clusters identified in cell-culture sample using two databases: cell type assigned by CellMarker (upper line) and by SingleCellBase (lower line); G2m_s npcs (neural progenitor cells); (**b**) GO Chord plot 1 cluster: GO:0051987 positive regulation of attachment of spindle microtubules to kinetochore, GO:0051256 mitotic spindle midzone assembly, GO:0051383 kinetochore organization, 2 cluster: GO:1905463 negative regulation of DNA duplex unwinding, GO:1902975 mitotic DNA replication initiation, GO:0000727 double-strand break repair via break-induced replication, GO:0051095 regulation of helicase activity (the full list of processes is presented in Table S4); (**c**) heatmap showing expression of the top genes associated with each cellular cluster (the full list of differentially expressed genes is presented in Supplementary Materials: Gbl27_heatmap.pdf).

The results of cell clustering for the Gbl27 sample are presented in Figure 6a. 

In the Gbl27 sample, three clusters practically merged and did not have well-defined borders. Monocytes 0 were characterized by expression of PLXDC2, CEBPD (regulator of immune and inflammatory responses), MSRB1 (regulator of actin assembly in macrophages), THBS1 (adhesive glycoprotein involved in inflammation and angiogenesis). Neural progenitor cells 1 expressed markers associated with mitosis, spindle assembly, and kinetochore organization (CCNB1, BIRC5, PRC1, DLGAP5, SMC4, and CENPF). In neural progenitor cells 2, the markers involved in DNA replication and repair were highly expressed (MCM2, TOP2A, MCM3, MCM4, MCM5, GINS2, MCM6, and MCM7) (Table S4) (Figure 6b,c; Supplementary Materials: Gbl27_info.xlsx). The trajectory started at the neural progenitor cells and moved to monocytes 0 and neural progenitor cells 2 (Figure S4).

When comparing the biological processes in different samples, it is noteworthy that, in the tumor, we observed processes related to nervous system cell differentiation (GO:0048714), nerve cell development and regeneration (GO:0031102, GO:0007399), cellular cytotoxicity (GO:0001788), and histocompatibility complex assembly (GO:0002503). 

In cell lines at early stages, the dominant processes were associated with cell adaptation to culturing conditions, including collagen metabolism (GO:0032963), blood-vessel development (GO:00015680), cholesterol biosynthesis (GO:0006695), and DNA replication and mitosis (GO:0006265, GO:0051256, and GO:0090267), in the Gbl17 or cellular responses to stress (HSA-2262752) in the Gbl13. In the Gbl27, practically all active processes were related to cell division and proliferation (GO:0051256, GO:0051383, and GO:1905463), as well as DNA replication and repair (GO:1902975, GO:0000727, and GO:0051095).

### 2.6. Cell Clustering of Gbl That Do Not Genetically Correspond to the Original Tumor Clone

In this section, we analyzed the glioblastoma cell-line samples that exhibited a mutational profile distinct from the primary tumor specimen. The cell lines have gone through varying numbers of passages. Gbl24 was at passage 7, and Gbl6 was at passage 9. The Gbl28 cell line derived from a patient with Grade 3 diffuse astrocytoma belongs to the same group, but it should be considered somewhat separately because this tumor has a lower degree of malignancy compared to GBM. Initially, the Gbl6, Gbl24, and Gbl28 samples contained 1054, 1285, and 640 cells, respectively. After filtration, it remained at 628, 864, and 415 cells in the corresponding samples (Table S2). 

**Figure 7:**
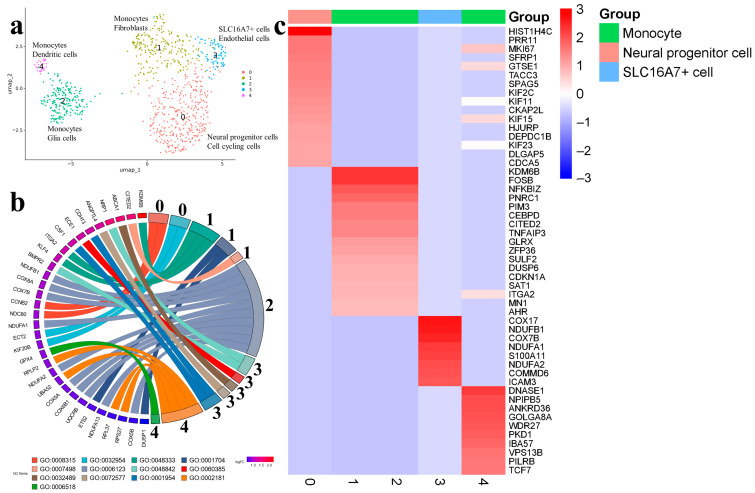
(**a**) Gbl24 (7 passage). In total, five cell clusters are identified in cell-culture sample using two databases: CellMarker (upper line) and SingleCellBase (lower line); (**b**) GO Chord plot 0 cluster: GO:0008315 G2/MI transition of meiotic cell cycle, GO:0032954 regulation of cytokinetic process, 1 cluster: GO:0048333 mesodermal cell differentiation, GO:0001704 formation of primary germ layer, GO:0007498 mesoderm development, 2 cluster: GO:0006123 mitochondrial electron transport, cytochrome c to oxygen, 3 cluster: GO:0048842 positive regulation of axon extension involved in axon guidance, GO:0060385 axonogenesis involved in innervation, GO:0032489 regulation of Cdc42 protein signal transduction, GO:0072577 endothelial cell apoptotic process, GO:0001954 positive regulation of cell-matrix adhesion, 4 cluster: GO:0002181 cytoplasmic translation, GO:0006518 peptide metabolic process (the full list of processes is presented in Table S5); (**c**) heatmap showing expression of the top genes associated with each cellular cluster (the full list of differentially expressed genes is presented in Supplementary Materials: Gbl24_heatmap.pdf).

Cell clustering in the sample Gbl24 revealed five clusters. Neural progenitor cells 0 had an expression of HIST1H4C (histone H4), PRR11 (transcription repressor regulating cell cycle), MKI67 (marker of proliferation Ki-67), KIF11 and KIF15 (microtubule binding and motor activity), GTSE1, NB2, and NDC80 (the G2/MI transition of the cell cycle) and some other markers involved in various DNA replication and regulatory processes (Figure 7, Table S5).

Monocytes were presented in three clusters. Monocytes 1 and 2 (KDM6B, FOSB, ITGA2, NFKBIZ, DUSP6) expressed cell markers of mesoderm development, indicating connective tissue formation and immune cell activity. Genes CDKN1A, CCNL1, and CSRNP1, common to both databases, are linked to the cyclin-dependent protein kinase holoenzyme complex. Monocytes 2 were enriched by the cell markers related to mitochondrial electron transport and oxidative phosphorylation, including NDUFA2, COX5B, and others (Figure 7b,c; Supplementary Materials: Gbl24_info.xlsx). Monocytes 4 (or dendritic cells according to SingleCellBase) expressed markers associated with mitochondrial electron transport and the respiratory electron transport chain. LGALS1, SNX3, and UBA52 were shared between two databases. The SLC16A7+ cells (or endothelial cells according to SingleCellBase) found in Cluster 3 differed from other clusters by expressing cell markers of the mitochondrial respiratory chain (COX17, NDUFB1, COX7B, NDUFA1, and NDUFA2), members of NF-kappa-B pathway (COMMD6), and immune response (ICAM3) (Figure 7c).

Single-cell trajectory analysis pointed to neural progenitor cells 0 as the starting point where actively dividing cells resided. Further, the trajectory evolved to monocytes 1, bifurcated to SLC16A7+ cells 3, and monocytes 2 (Glial cells) continued to monocytes 4 (dendritic cells) (Figure S5).

**Figure 8:**
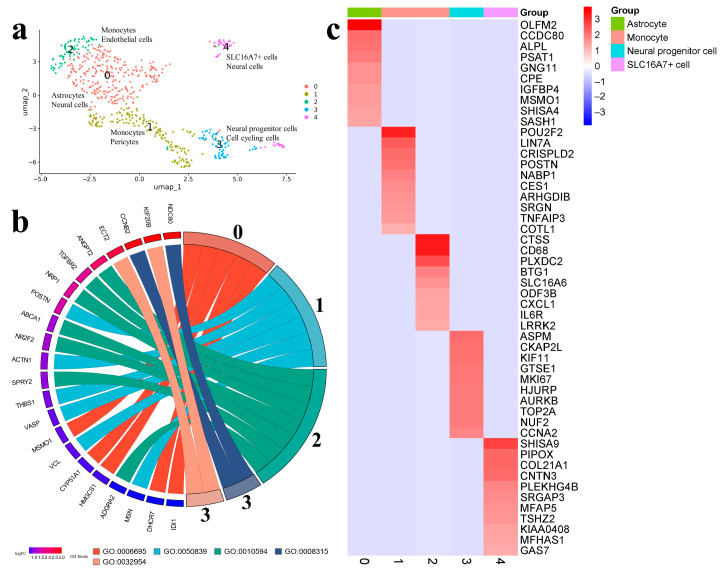
(**a**) Gbl6 (9 passage). Cell clusters identified in cell-culture sample using two databases: CellMarker (upper line) and SingleCellBase (lower line); (**b**) GO Chord plot 0 cluster: GO:0006695 cholesterol biosynthetic process, 1 cluster: GO:0050839 cell-adhesion molecule binding, 2 cluster: GO:0010594 regulation of endothelial cell migration, 3 cluster: GO:0008315 G2/MI transition of meiotic cell cycle, GO:0032954 regulation of cytokinetic process (the full list of processes is presented in Table S5); (**c**) heatmap showing expression of the top genes associated with each cellular cluster (the full list of differentially expressed genes is presented in Supplementary Materials: Gbl6_heatmap.pdf).

A wider diversity of cell types was found in the Gbl6 sample (Figure 8a). 

Astrocytes 0 expressed DHCR7, HMGCS1, CYP51A1, IDI1, and MSMO1, which are involved in the cholesterol biosynthetic process; and CCDC80, and PSAT1, which are participated in L-serine biosynthesis. ALPL, an alkaline phosphatase, and GRIA1, a glutamate receptor, were markers common to both databases. The monocytes formed two clusters. Monocytes 1 (or pericytes) expressed the cell-adhesion markers VCL, COTL1, ACTN1, VASP, MSN, THBS1, and POSTN. Monocytes 2 (or endothelial cells) were characterized by a higher expression of markers related to the immune system or angiogenesis, including CD68, LGALS3, IL6R, CTSS, LRRK2, CXCL1, FAM49A, ANGPT2 and ADGRA2 (Figure 8b,c).

Neural progenitor cells 3 were characterized by the expression of CCNB2, NDC80, EST1, KIF20B, KIF11, MK167, NUF2, TOP2A, AURKB, and CCNA2, which are mainly involved in cell-cycle regulation, chromosome segregation during mitosis, and DNA replication and repair. The SLC16A7+ cells were assigned to cluster 4 with markers SHISA9 (regulator of short-term neuronal synaptic plasticity), PIPOX (peroxisomal enzyme) COL21A1, CNTN3 (cell adhesion and nervous system development), SRGAP3 (negative regulator of cell migration), and a shared marker AKAP9 (scaffolding protein crucial for the integrity of the Golgi apparatus and the mitotic spindle during metaphase) (Figure 8b,c; Table S5; Supplementary Materials: Gbl6_info.xlsx).

A single-cell trajectory started at neural progenitor cells 3, then led to monocytes 1 and astrocytes 0. Another branch of the trajectory transited to monocytes 2 and then to the SLC16A7+ cells 4 (Figure S6).

**Figure 9:**
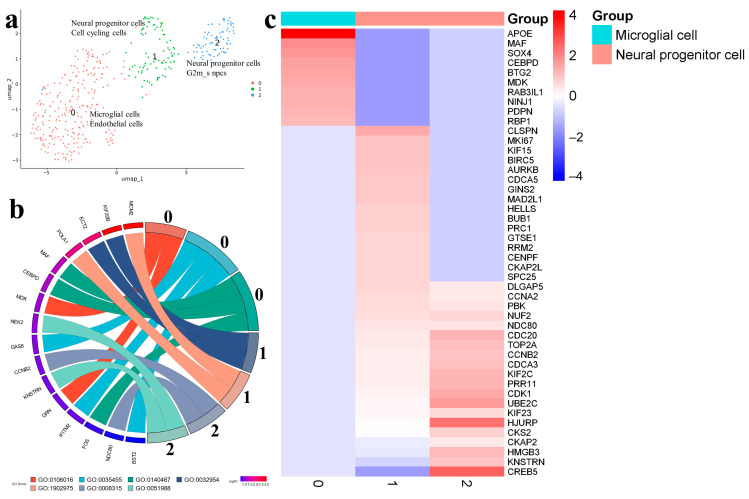
(**a**) Gbl28 (5 passage). Cell clusters identified in cell-culture sample using two databases: cell type assigned by CellMarker (upper line) and by SingleCellBase (lower line); G2m_s npcs (neural progenitor cells); (**b**) GO Chord plot 0 cluster: GO:0106016 positive regulation of inflammatory response to wounding, GO:0035455 response to interferon-alpha, GO:0140467 integrated stress-response signaling, 1 cluster: GO:0032954 regulation of cytokinetic process, GO:1902975 mitotic DNA replication initiation, 2 cluster: GO:0008315 G2/MI transition of meiotic cell cycle, GO:0051988 regulation of attachment of spindle microtubules to kinetochore (the full list of processes is presented in Table S5); (**c**) heatmap showing expression of the top genes associated with each cellular cluster (the full list of differentially expressed genes is presented in Supplementary Materials: Gbl28_heatmap.pdf).

The Gbl28 sample (five passages) contained only three clusters (Figure 9a). Microglial cells 0 (or endothelial cells) expressed markers associated with inflammatory processes (GRN and MDK), the response to interferon-alpha (BST2 and IFITM2), integrated stress-response signaling (FOS, MAF, and CEBPD), the regulation of angiogenesis NINJ1, and the blood vessel development PDPN (Table S5).

The markers of neural progenitor cells 1 and 2 were linked to the G2/MI transition of the cell cycle (NDC80 and CCNB2). The shared markers between the two databases included CKAP2, CKS2, KNSTRN, NUF2, PBK, and TOP2A (Figure 9b,c; Supplementary Materials: Gbl28_info.xlsx).

When analyzing trajectories, neural progenitor cells 2 can be considered the starting point. The trajectory then proceeded to neural progenitor cells 1 and microglial cells 0 (Figure S7).

The dominant biological processes in this group of samples were related to cell division (GO:0008315, GO:0032954), the regulation of cytokinetic process, mesodermal cell differentiation (GO:0048333) and mesoderm development (GO:0007498), mitochondrial electron transport ( GO:0006123), endothelial cell apoptotic process (GO:0072577), cell-matrix adhesion (GO:0001954), and the regulation of immune processes (GO:0106016, GO:0035455).

## 3. Discussion

A deeper understanding of inter- and intratumor heterogeneity in glioblastoma has largely become possible by the development of single-cell sequencing approaches. This provides new insights into the molecular organization of the tumor and elucidates the role of heterogeneity in the development of drug resistance and tumor recurrence [18].

Two layers of glioblastoma heterogeneity are considered. It is suggested that the first layer is represented by different transcriptional subtypes: proneural (TCGA-PN), classical (TCGA-CL), mesenchymal (TCGA-MES), and neural (TCGA-N) [8,13]. The second layer of heterogeneity is related to the developmental state of glioblastoma cells within the tumor. Glioblastoma mimics the developmental mechanisms of brain cells and contains subsets of glioblastoma stem cells (GSCs). GSCs are speculated to generate new tumor clones with pre-mature resistance to radiation and chemotherapy [19,20,21]. It is assumed that GSC subpopulations can have different sets of transcribed markers and generate tumor clones that differ in cell composition and directions of development [22,23]. 

While TCGA’s classification of glioblastoma into four distinct molecular subgroups aimed to address the issue of tumor heterogeneity, recent studies using single-cell technologies show that the subgroups are flexible and can vary spatially and temporally within a single tumor. It was shown that a single tumor exhibited features of all four subtypes [12,24]. Tumor cells in glioblastoma interact with the tumor microenvironment, forming a complex environment that stimulates the transcriptional adaptation of tumor cells and promotes disease progression [13,25].

In the present study, we investigated the diversity of cell types in a single glioblastoma tumor sample and six patient-derived cell lines and identified the most characteristic biomarkers whose expression might reflect key cell characteristics and influence cell behavior. Four types of samples were analyzed: (1) intraoperative glioblastoma (Gb75t); (2) patient-derived glioblastoma cell lines with a conserved mutational profile from the original tumor (Gbl17 at passage 3, Gbl15 at passage 5, and Gbl27 at passage 11); (3) patient-derived glioblastoma cell lines that differed from the original tumor by their mutations (Gbl24 at passage 7 and Gbl6 at passage 9); and (4) a patient-derived cell line established from a tumor classified as an IDH-wildtype astrocytoma, Grade 3 (Gbl28 at passage 5). The passage count was determined at each cell-culture reseeding when 70–80% confluency was achieved. We assumed that, up to ten passages, a cell culture can change its cellular composition, and thereafter, it becomes more defined. According to the established opinion in the literature, a cell line can be considered established when it has undergone 8–10 passages [26]. 

Cells in scRNA-seq datasets are commonly classified as either malignant or normal based on the presence or absence of CNVs, respectively [27]. An analysis of CNVs in the studied samples showed that, in all cases, the cells had a rearranged genome compared to normal brain tissue cells (Figure 1) Thus, even in cases where the cell lines did not contain driver mutations inherent to the original tumor (e.g., Gbl24), we were dealing mostly with transformed cells with potential proliferative characteristics of malignant growth. Also, the integrated pattern of the transcriptional profiles of cells in all the samples mostly differed from the transcriptional profile of normal brain tissue (Figure 2). 

The Gb75t tumor sample represented a heterogeneous mixture of well-defined cell populations and included several types of astrocyte-like cells, SLC16A7+ or neural-like cells, and tumor microenvironment cells (micro- and macroglia, dendritic cells, and regulator T-lymphocytes). A similar cellular composition has been previously described in other studies on the scRNA-seq of glioblastoma specimens [12,16,24]. Also, non-malignant cells identified as astrocytes were presented. Due to the diffuse growth of glioblastoma and the invasion of cancer cells into the brain parenchyma, operative specimens often may contain regions from the peritumoral brain zone. It was shown that the peritumoral brain zone, having the radiological characteristics of normal brain tissue, nevertheless contains specific tumor and stromal cells that promote glioblastoma growth and invasion [28].

In the Gbl17 (3 p), mesenchymal-like cells and astrocytes prevailed in the total sample; SLC16A7+ cells were missing. Microenvironmental cells were presented by glial cells and proliferated monocytes. In the Gbl13 sample (5 p), SLC16A7+ (or mesenchymal-like) cells and FOXN4+ cells were the major subpopulations of tumor cells, while neuronal cells and astrocytes were missing. In the Gbl27 sample (11 p), only actively proliferating neural cell precursors and monocytes were found.

Most genes from the cell populations of the samples contributed to the malignant phenotype of glioblastoma and its high invasiveness. Expression of the SLC16A7 gene was observed in the tumor sample, as well as in patient-derived cell lines Gbl13 and Gbl17. SLC16A7 is a coding high-affinity pyruvate transporter MCT2, which was found to be expressed by endothelial cells delineating cerebral blood vessels [29]. Tumors produce an increased amount of lactate, which is expelled from the cells by monocarboxylate transporters (MCTs). MCT2 appears to be predominantly present in neurons, where it is found on axons and dendrites, with a particular enrichment in dendritic spines. Axonal uptake of glycolysis products via MCT2 supports mitochondrial energy metabolism and, thus, may promote tumor growth and progression [30]. The *FOXN4* gene expression is elevated in the Gbl13 sample subpopulation of progenitor cells. FOXO4 is a pleiotropic transcription factor involved in the regulation of a wide range of cellular pathways essential for cell survival, and thus associated with malignant cell transformation and tumorigenesis [31]. 

It should be noted that these three cell lines had driver mutations in the *PTEN* gene. In addition, the loss of *PTEN* heterozygosity was observed in these cell lines. A common observation for this group of samples was a decrease in heterogeneity from early passages (Gbl13 and Gbl17) to later passage (Gbl27) and a relative substitution of differentiated cell types, like astrocytes, by the populations of actively proliferating neural progenitor cells. The following dominant biological processes can be noted within these samples across various passages. Early passages were marked by vascular development, the cholesterol biosynthesis that is essential for gliogenesis and antioxidant defense mechanisms (the Gbl17); or by cellular responses to stress stimuli and the organization of collagen fibrils (the Gbl13). In the Gbl27 (11 passage), the dominating processes were related to the cytoskeleton, cellular division, translation, and the cell’s energy supply.

Genetically distinct from the original tumor samples, the Gbl6 and Gbl24 showed a different cellular composition. In both samples, several monocyte clusters with distinct functions were revealed. Monocytes 1 and 2 in the Gbl24 sample showed involvement in immune response and cell-adhesion processes, as indicated by the expression of markers such as KDM6B, ITGA2, and VCL. Consistent with our results, ITGA2 was found to be significantly upregulated in both the glioblastoma tumor tissues and cell lines, with minimal expression in normal tissues. Furthermore, antibody blockade of ITGA2 potently inhibits glioblastoma cell migration [32]. Monocytes 2 and 4 in Gbl24 and monocytes 2 in Gbl6 are involved in mitochondrial electron transport and oxidative phosphorylation, which are crucial for energy production. These findings suggest that monocytes within the tumor microenvironment not only support immune functions but also contribute to the metabolic adaptations necessary for tumor survival. The Gbl28 sample, derived from a Grade 3 diffuse astrocytoma, showed low cell type diversity among patient-derived cell lines, having only neural progenitor cells and microglial cells (or endothelial cells according to SingleCellBase) involved in inflammation, response to interferon-alpha, and integrated stress-response signaling. 

Interestingly, all Gbl samples without driver mutations of the original tumor (Gbl6, Gbl24, and Gbl28) included clusters of endothelial cells, as they were identified by using the SingleCellBase database. In the brain, endothelial cells are the main cellular component of the blood–brain barrier. Endothelial cells regulate vascular permeability and angiogenesis, and vascular dysfunction and active proliferation are the most characteristic features of glioblastoma and contribute to glioblastoma tumor cell invasion [33]. In the Gbl6, the cell typing with SingleCellBase revealed another important cell population of pericytes (cluster monocytes 1 or pericytes). Pericytes represent mural cells located between the endothelial cells of capillaries and the basement membrane, which maintain normal blood microcirculation in tissues. In glioblastoma, pericytes represent one of the important cell populations in the tumor microenvironment [34]. Pericytes and tumor cells may interact to secrete proteins, nucleic acids, or extracellular vesicles; in addition, transformed cancer cells may exhibit the phenotype and functions of pericytes, accumulate in local blood vessels, and induce new foci of tumor growth [35]. One of the markers highly expressed in cluster 1 of the Gbl6 was POSTN. The gene *POSTN* encodes the matricellular protein periostin, which is associated with glioma progression. It was shown that periostin expression in gliomas is related to neo-angiogenesis, and the expression is most significant in gliomas with microvascular proliferation (glioblastoma) [36]. 

The absence of specific mutations inherent to the original tumor in samples of glioblastoma cell lines (Gbl6, Gbl24, Gbl28) may be explained by the proliferation of selected cell populations (for example, from the tumor microenvironment) depleted by the presence of cancer cells with corresponding mutations. Another explanation may be related to the phenomenon of tumor subclonality. In another study, two distinct cancer cell lines with different morphological and biological characteristics were successfully isolated and established from the same tissue sample of a glioblastoma [37]. In any case, these cell lines are presented by transformed cells with evidence of malignant growth, which are characterized by rearranged genomes and corresponding expression patterns of tumor cells.

When identical cell types were identified in different samples, the biological processes in them differed, suggesting that these cells undergo a change in transcriptional profile in response to replicative senescence and microenvironmental changes in vitro. We revealed the transcriptomic heterogeneity of astrocytes across the samples (Gb75t, Gb17, and Gb6), as well as within one sample (Gb75t and Gbl17). In the Gbl6 sample, astrocytes in cluster 0 expressed genes involved in the cholesterol biosynthetic process, which points to a role of lipid metabolism in glioblastoma, that may contribute to membrane synthesis, signaling pathways, and cellular proliferation. Similar observations were made earlier [38]. 

The presence of a subpopulation of actively proliferating neural progenitor cells should also be noted in the Gbl27, Gbl24, and Gbl28 samples. The cell subtype was characterized by the expression of NDC80 and CCNB2 associated with G2/MI cell-cycle transition and the expression of NP32E, ATAD2, FEN1, HIST1H4C, KIF23, TOP2A, MCM2, MCM6, GINS2, etc., mainly involved in DNA replication and repair. 

Almost all cell-culture sample processes related to the regulation of molecular oxygen availability were revealed, including GO:0001568 blood-vessel development; GO:0045765 regulation of angiogenesis; GO:0010273 detoxification of copper ion; GO:0006119 oxidative phosphorylation; GO:0006123 mitochondrial electron transport, cytochrome c to oxygen; GO:0019646 aerobic electron transport chain; GO:0042775 mitochondrial ATP synthesis coupled electron transport; GO:0022904 respiratory electron transport chain; and GO:0035794 positive regulation of mitochondrial membrane permeability (Tables S3–S5).

Cell typing currently presents a complex challenge. Among the existing methods, typing by specific markers, typing by biological processes, and label transferring are the most commonly used. One of the main limitations of typing is the absence of specific marker genes in the list of differentially expressed (DE) genes. In such cases, it must be acknowledged that the cells in the culture are in transitional forms and lack their markers due to degradation or internalization.

The choice of marker database is also of great significance. When creating databases, datasets from various tissues and organs are used, so it is essential to ensure that the database contains datasets of the tissue under analysis [39,40].

Interestingly, even when using several databases containing datasets of the required tissue, the results can vary. This is due to the disruption of the proportions of markers represented for different cell types. For instance, if there are many markers for one cell type, the likelihood that a cell will be identified as that type is very high. Therefore, it is necessary to analyze not only the first determined type but also several subsequent ones and compare these results with typing by other databases, where the same cell type may be positioned at adjacent ranks [41].

The first limitation of this study is that we did not have the opportunity to trace the path of a single sample from the original tumor to the cell culture taken at different passages. This is due to the peculiarities of cell suspension preparation for the sequencing of single cells since we worked with living cells, and the library preparation was performed at one time. In this work, we tried to avoid cryopreservation, since the procedure might affect the original cellular composition of the samples. Subsequently, all these cell cultures were frozen and successfully thawed, which makes it possible to use them further in experiments. The second limitation is that we cannot count the number of divisions made by individual cells very accurately. The number of cell transitions or generations was estimated with a certain degree of assumption. Nevertheless, this allowed for distinguishing between cultures in early passages and more mature cell lines. The third limitation is that, at present time, there is no single algorithm for the most accurate determination of cellular subtype. The need to use different algorithms and find a consensus between them does not make it possible to unambiguously determine the type of cells and the prevailing biological processes. 

Taken together, our results provide a foundation for understanding the cellular and molecular mechanisms underlying glioblastoma heterogeneity, paving the way for the development of targeted therapies aimed at disrupting critical pathways involved in tumor maintenance and progression.

## 4. Materials and Methods

### 4.1. Tissue Collection and Cell Culture

Surgical material from macroscopically altered glioblastoma tumor tissue was used, more often involving the peritumoral zone in the operative area. Immediately after resection, a piece of tumor 0.5–2 cm^3^ in size was placed in DMEM medium (Gibco, Miami, FL, USA) and transported to the laboratory for 2–12 h. The piece was washed with Versene solution (PanEco, Moscow, Russia), and congealed blood, visible vessels, and burned areas were removed. The tumor tissues were minced with a scalpel and subsequently incubated in 0.25% Trypsin solution (PanEco, Moscow, Russia) for 10–30 min to obtain a cell suspension. Next, homogenized cell suspension was transferred into T25 cell-culture flasks (Corning Costar, New York, NY, USA) containing DMEM/F12 culture medium (Gibco, Miami, FL, USA) supplemented with 7% fetal bovine serum (HyClone, Logan, UT, USA), 100 U/mL penicillin and streptomycin, and 2 mM L-glutamine (PanEco, Moscow, Russia). The cells were cultured and maintained as adherent cultures at 37 °C in a humidified atmosphere with 5% CO_2_. When the cells reached 70–90% confluency, they were detached from the surface by 0.25% Trypsin solution (PanEco, Moscow, Russia). The suspension was diluted 2–3 fold and moved to a new flask. We counted as one passage every such reseeding of the cell culture. 

### 4.2. DNA Isolation and Multigene Panel Testing

DNA was isolated from fresh-frozen tumor tissue, cultured cells, or leukocytes from peripheral blood using the QIAamp kit DNA Mini Kit (Qiagen, Hilden, Germany). Libraries were prepared using the KAPA HyperPrep Kit (Roche, Basel, Switzerland) following the manufacturer’s protocol. Prepared libraries were subjected to hybridization with a custom panel of coding regions of 812 cancer-associated genes following the Hyper protocol (Roche, Basel, Switzerland). Sequencing was performed on the NextSeq2000 platform from Illumina (Illumina, San Diego, CA, USA) using a paired-end protocol with 200 cycles, achieving up to 500× coverage.

### 4.3. Cell Preparation for Single-Cell Analysis

Cell preparation was conducted according to the 10× Genomics single cell protocol (10× Genomics, Pleasanton, CA, USA). Briefly, the cell suspension was centrifuged at 300 rcf for 5 min, and the cell pellet was washed in 1 mL of 1× PBS with 0.04% BSA (Thermo Fisher Scientific, Waltham, MA, USA) two times. Then, a cell strainer 40 mkm (Thermo Fisher Scientific, Waltham, MA, USA) was used to remove any remaining debris or large clumps, and the cells at a final concentration of 2000 cells/µL were used for further analysis. The total cell count was 10,000 cells per reaction.

### 4.4. Library Preparation and Sequencing

A Chromium Next GEM Single cell 3’ Reagent kit v3.1 (10× Genomics, Pleasanton, CA, USA) was used to prepare scRNA-seq libraries following the manufacturer’s instructions. Briefly, the suspension of single cells was loaded into the Chromium Single Cell Controller Instrument (10× Genomics) to generate Gel Beads-in-emulsion (GEMs). The cell lysis and barcoded reverse transcription of RNA were performed, followed by the disruption of emulsions using the recovery agent. DynaBeadsMyOne Silane Beads (Thermo Fisher Scientific, Waltham, MA, USA) were used to clean the cDNA. The cDNA was amplified, and the final libraries of the individual samples were evaluated on the Agilent Bioanalyzer using a High Sensitivity DNA Kit (Agilent Technologies, Santa Clara, CA, USA). All libraries were pooled together and initially sequenced on an Illumina NextSeq 2000 (Illumina, San Diego, CA, USA) with flow cell using v2.5 chemistry and paired-end sequencing with single indexing following Illumina protocols and 10× sequencing parameters (28 bp Read 1, 8 bp i7 index, 91 bp Read 2).

### 4.5. Raw Data Preprocessing

The conversion of BCL files to FASTQ format was performed using the Illumina BCL Convert Dragen platform. Secondary analysis of the FASTQ data was conducted using the Illumina DRAGEN Bio-IT Platform v2.6.5. The resulting files, including barcodes, barcode summary, features, and matrix, were utilized for further analysis.

### 4.6. Preprocessing and Cells Clustering

For further analysis, the barcode, barcode summary, features, and matrix files were imported into Seurat (version 5.0.3) R (version 4.2.3). Low-quality cell filtration was conducted based on the parameters presented in Table S1.

Normalization, scaling, identification of highly variable genes, dimensionality reduction, clustering, and visualization of the results were performed using the Seurat package. The clustering utilized a resolution parameter of 0.4.

The identification and removal of cell doublets were carried out using the DoubletFinder package (version 2.0.4) [18]. The number of cells before filtration and the number of identified doublets and singlets are presented in Table S2.

### 4.7. CNV

The determination of copy-number variations (CNVs) was performed using the Infer CNV library, designed to detect somatic large-scale chromosomal copy-number changes (or large chromosomal segments) in single-cell tumor RNA-seq data by analyzing the gene-expression intensity across the entire genome of tumor cells compared to a control set of benchmark “normal” cells GSE157827 (GSM4775574).

### 4.8. Cell Typing

Lists of differentially expressed genes for each cluster were obtained using the FindAllMarkers function of the Seurat package. The lists were exported in CSV format. For cell-type identification, the CellMarker DB and singleCellBase marker databases were used. The DE gene lists were matched with the marker lists from the CellMarker and singleCellBase databases, after which the number of matches between these lists was calculated. The resulting list was then ranked, and the identified cells were assigned the cell type for which the greatest number of matches was found.

### 4.9. Generation of Heatmap and GO Chord Plot

To generate heatmaps, the R package SRplot was utilized. Heatmaps were constructed using lists of differentially expressed genes (DEGs) (with avg_log2FC > 0.6) extracted from Excel files, which are provided in the Supplemental Materials for each sample. For the abbreviated versions of the heatmaps included in the figure panels for each sample, only the top 5–10 genes were added. The full versions of the heatmaps included all marker genes used for classification.

GO Chord plots were generated using the R package SRplot as well. Marker genes belonging to specific gene ontology (GO) groups were used to construct GO chord plots. The online services STRING and g:Profiler were employed to determine membership in specific GO categories. 

### 4.10. Integration

For integration, the canonical correlation analysis (CCA) method of the IntegrateLayers function of the Seurat package was used. Clustering was performed using the FindNeighbors and FindClusters functions. Subsequently, the algorithm for modularity optimization with a resolution argument value of 0.4 was applied.

### 4.11. Trajectory Inference

In this study, the Slingshot and Monocle3 methods were employed for the construction of developmental trajectories. For trajectory analysis in Monocle3, a Seurat object was transformed into a monocle object, preserving the original UMAP coordinates, followed by Louvain clustering. Subsequently, a trajectory was constructed, with start cells designated as priors. Aligning cells along the trajectory facilitated the derivation of pseudotime values for each cell. For trajectory construction in Slingshot, counts, clustering, and UMAP from the Seurat Object were utilized.

## 5. Conclusions

In summary, we demonstrate that patient-derived cell lines of glioblastoma are characterized by heterogeneity, and it can be assumed that the diversity of cellular populations depends on the spatial localization of the original source of cells and possibly on the duration of cultivation. In some cases, we have an unpredictable set of clones from the patient’s operative material, including the cancer cells themselves and different cells from the tumor microenvironment and peritumoral brain zone. In our research, the cell lines with a conserved genome of the original tumor showed a tendency to transition from a high diversity of cell subtypes to more narrow spectra of dedifferentiated cells during cultivation. The cell lines with a genome different from the original tumor refer to cells with signs of malignancy according to CNV analysis and integrated transcriptional profiles. The cell lines from this group of samples are enriched by cell populations referred to as the tumor microenvironment. Thus, single-cell RNA-sequencing cancer research has enormously contributed to deciphering the exact cellular composition of tumor and cell-culture specimens. The different cell types coexisting in a tumor, as well as the molecular diversity of individual cells, form a complex ecosystem that interferes with treatment efficacy. The cell-culture models partially retained intratumor heterogeneity but could also have their specific characteristics, which is important to consider in translational studies. The identification of clinically relevant cell subpopulations (highly proliferative, aggressively migrating, immunosuppressive, etc.) can be considered as an important direction in the search for new biomarkers and therapeutic targets.

## Figures and Tables

**Table 1:** Characteristics of scRNA-seq samples (GBM—glioblastoma, DA3—diffuse astrocytoma, Grade 3, Gb75t—glioblastoma tumor sample from patient #75, Gbl—patient-derived glioblastoma cell line).

ID	Passage	Mutated Genes in Cell Line	VAF, %	Patient’s Age, Sex	Diagnosis	Mutated Genes in Original Tumor	VAF, %
Gb75t	-	-	-	58, m	GBM	*TERT*	53
						*CDKN2A*	50
						*PIK3C2B*	48
Gbl27	11	*PTEN*	100	57, m	GBM	*PTEN*	56
		*TP53*	100			*EGFR*	48
Gbl13	5	*PTEN*	99	45, m	GBM	*PTEN*	56
		*TP53*	100			*TP53*	72
						*TERT*	30
Gbl17	3	*PTEN*	95	67, m	GBM	*PTEN*	50
		*ERBB3*	36			*ERBB3*	16
Gbl6	9	*CD177*	31	64, m	GBM	*SEC24D*	48
		*FAM120B*	32			*IKBKB*	26
Gbl24	7	*PIM1*	43	49, m	GBM	*TERT*	36
						*PTEN*	76
						*TP53*	38
Gbl28	5	-	-	46, m	DA3	*EGFR*	19

## Data Availability

Data are available on request.

## Supplementary Materials

The following supporting information can be downloaded at https://www.mdpi.com/article/10.3390/ijms25158472/s1.
