# Supplementary material for: Intratumoral Cell Heterogeneity in Patient-Derived Glioblastoma Cell Lines Revealed by Single-Cell RNA-Sequencing
Source: Int J Mol Sci. 2024 Aug 2;25(15):8472. doi: 10.3390/ijms25158472 (PMC11313325; doi:10.3390/ijms25158472)
Supplement: Supplementary file 1 [file ijms-25-08472-s001.zip › Gbl27_heatmap.pdf]

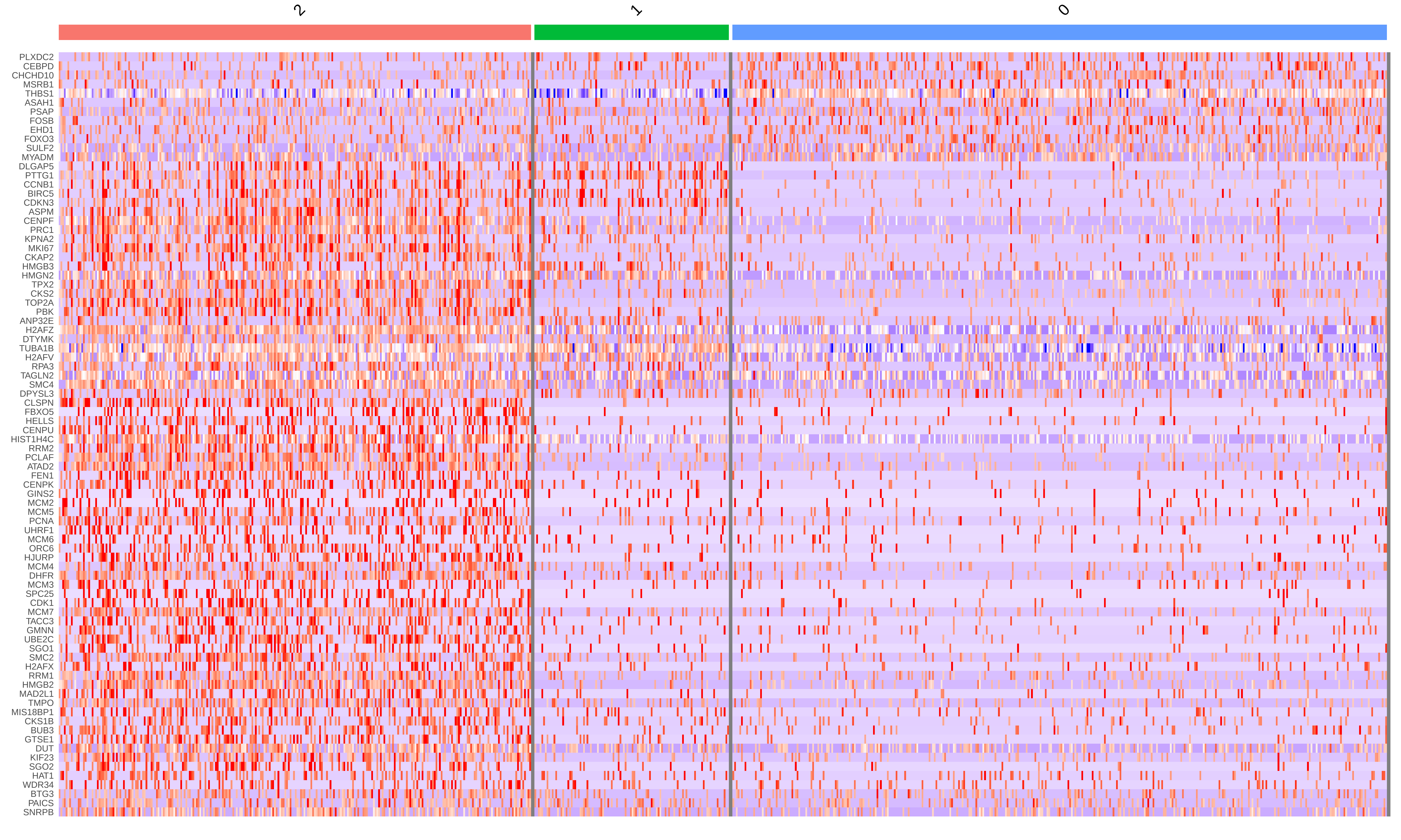

2
1
0
PLXDC2
CEBPD
CHCHD10
MSRB1
THBS1
ASAH1
PSAP
FOSB
EHD1
FOXO3
SULF2
MYADM
DLGAP5
PTTG1
CCNB1
BIRC5
CDKN3
ASPM
CENPF
PRC1
KPNA2
MKI67
CKAP2
HMGB3
HMGN2
TPX2
CKS2
TOP2A
PBK
ANP32E
H2AFZ
DTYMK
TUBA1B
H2AFV
RPA3
TAGLN2
SMC4
DPYSL3
CLSPN
FBXO5
HELLS
CENPU
HIST1H4C
RRM2
PCLAF
ATAD2
FEN1
CENPK
GINS2
MCM2
MCM5
PCNA
UHRF1
MCM6
ORC6
HJURP
MCM4
DHFR
MCM3
SPC25
CDK1
MCM7
TACC3
GMNN
UBE2C
SGO1
SMC2
H2AFX
RRM1
HMGB2
MAD2L1
TMPO
MIS18BP1
CKS1B
BUB3
GTSE1
DUT
KIF23
SGO2
HAT1
WDR34
BTG3
PAICS
SNRPB
