# Supplementary material for: Intratumoral Cell Heterogeneity in Patient-Derived Glioblastoma Cell Lines Revealed by Single-Cell RNA-Sequencing
Source: Int J Mol Sci. 2024 Aug 2;25(15):8472. doi: 10.3390/ijms25158472 (PMC11313325; doi:10.3390/ijms25158472)
Supplement: Supplementary file 1 [file ijms-25-08472-s001.zip › Supplementary materials.pdf]

Supplementary materials

Table S1. Cell filtration parameters

Sample	nFeature_RNA	nCount_RNA	percent.mt
Gbl6	>2500<10000	<60000	<10
Gbl13	>2500<8000	<60000	<10
Gbl17	>1750<3500	<10000	<12.5
Gbl24	>1500<8500	<65000	<8
Gbl27	>1000<3000	<6000	<6
Gbl28	>3000<10000	<100000	<10
Gb75t	>500<4000	<12000	<15

Table S2. Initial number pf cells, doublet and singlet number

Sample	Initial number of cells	Doublet	Singlet
Gbl6	1054	32	628
Gbl13	829	34	581
Gbl17	4590	126	2348
Gbl24	1285	107	864
Gbl27	1626	42	861
Gbl28	640	18	415
Gb75t	1891	80	1192

Sample of intraoperative tumor material (glioblastoma)

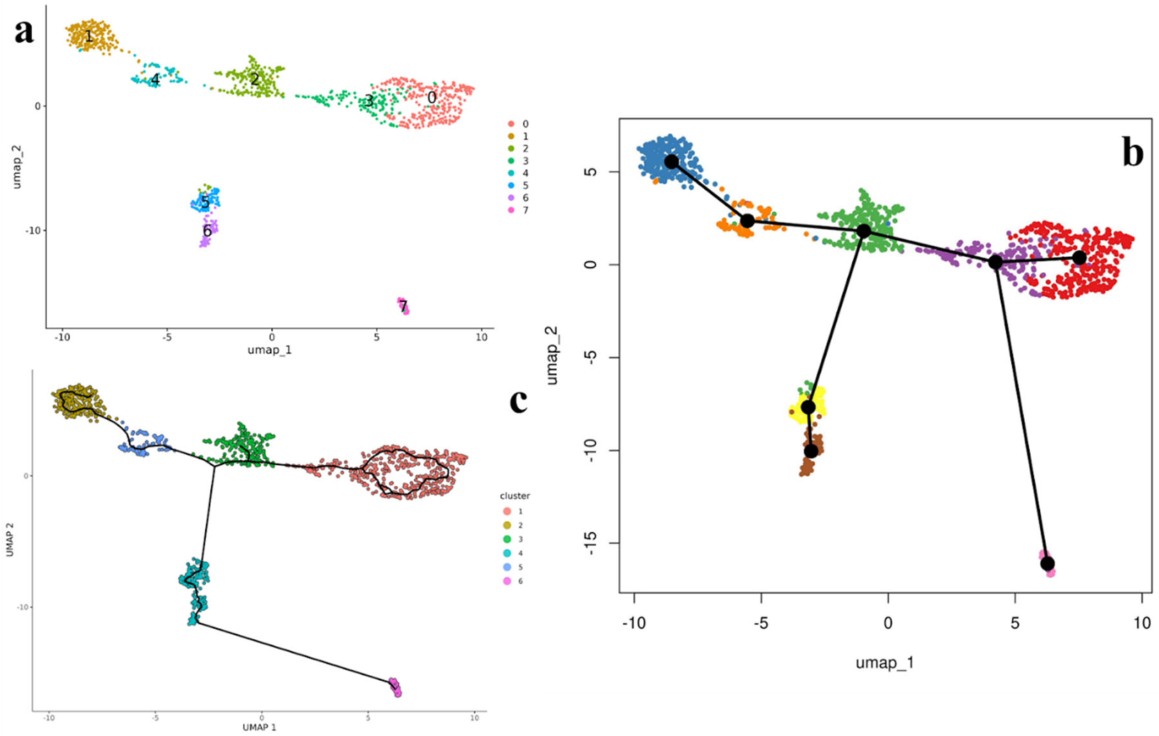

Figure S1. **Gb75t** Glioblastoma sample analysis. (**a**) Clusters (**CellMarker DB** 0 **-** Microglial cells, 1 - Astrocytes, 2 - Astrocytes, 3 - Microglial cells, 4 - SLC16A7+ cells, 5 - SLC16A7+ cells, 6 - Astrocytes, 7 - Monocytes. **singleCellBase** 0 - Monocytes. 1 - Neural cells, 2 - Glia cells, 3 - Dendritic cells, 4 - Neural cells, 5 - Neural cells, 6 - Neural cells, 7 - T cells); (**b**) Slingshot trajectory; (**c**) Monocle trajectory on clusters

Table S3. Gb75t sample cell types and biological process

Cluster	GO (Biological process)	Common markers (CellMarker DB, singleCellBase)
0 (Microglial cell, Endothelial cells)	GO:0106016 Positive regulation of inflammatory response to wounding<br>GO:0035455 Response to interferon-alpha<br>GO:0140467 Integrated stress response signaling	no
1 (Neural progenitor cell, Cell cycling cells)	GO:0008315 G2/MI transition of meiotic cell cycle<br>GO:0032954 Regulation of cytokinetic process<br>GO:1902975 Mitotic DNA replication initiation	no
2 (Neural progenitor cell, G2m_s npcs)	GO:0008315 G2/MI transition of meiotic cell cycle<br>GO:0051988 Regulation of attachment of spindle microtubules to kinetochore	CKAP2, CKS2, KNSTRN, NUF2, PBK, TOP2A
3 (Glia cells)	GO:0001788 Antibody-dependent cellular cytotoxicity	CXCL16, FCGR3A, MIS18BP1, MS4A7, RASSF4
4 (Neural cells, SLC16A7+ cells)	GO:0048024 Regulation of mRNA splicing, via spliceosome	AKAP9
5 (Neural cells, SLC16A7+ cells)	no	ENO2, WSB1
6 (Neural cells, Astrocytes)	GO:0007399 Nervous system development	DGKG, FERMT2, NFIA
7 (Monocytes, T-cells)	GO:1905686 Positive regulation of plasma membrane repair<br>GO:0002250 Adaptive immune response	CARD16, LSP1, S100A4

Cell cultures that harbor the same mutations as their originating tumors, thereby mirroring their tumors at a genetic level

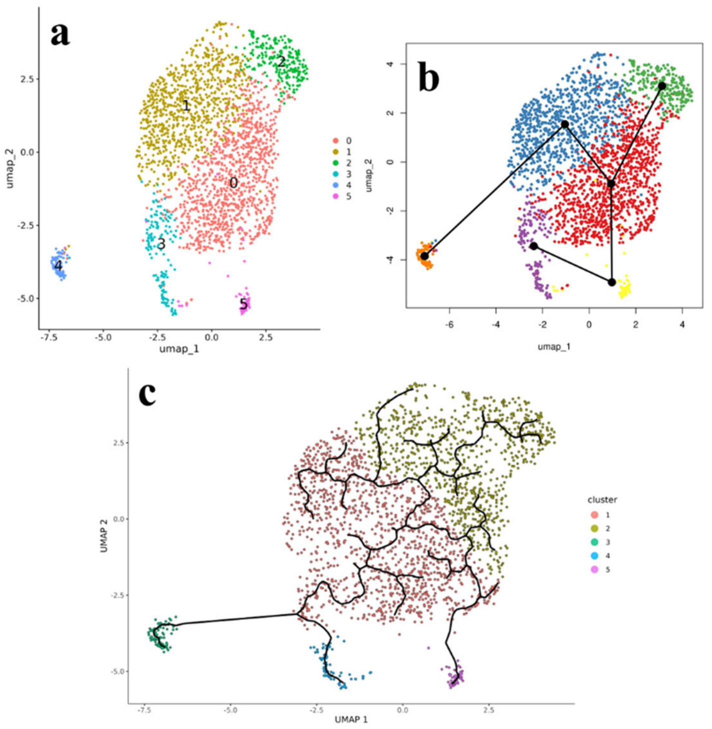

Figure S2. **Gbl17 (3 passage)** Glioblastoma sample analysis. (**a**) Clusters (**CellMarker DB** 0 - DCLK1+ progenitor cells, 1 - Astrocytes, 2 - Astrocytes, 3 - Monocytes, 4 - Monocytes, 5 - Astrocytes. **singleCellBase** 0 - Mesenchymal-like cells, 1 - Fibroblasts-like cells, 2 - Erythrocytes, 3 - G2m_s npcs, 4 - Glia cells, 5 - Monocytes); (**b**) Slingshot trajectory; (**c**) Monocle trajectory on clusters

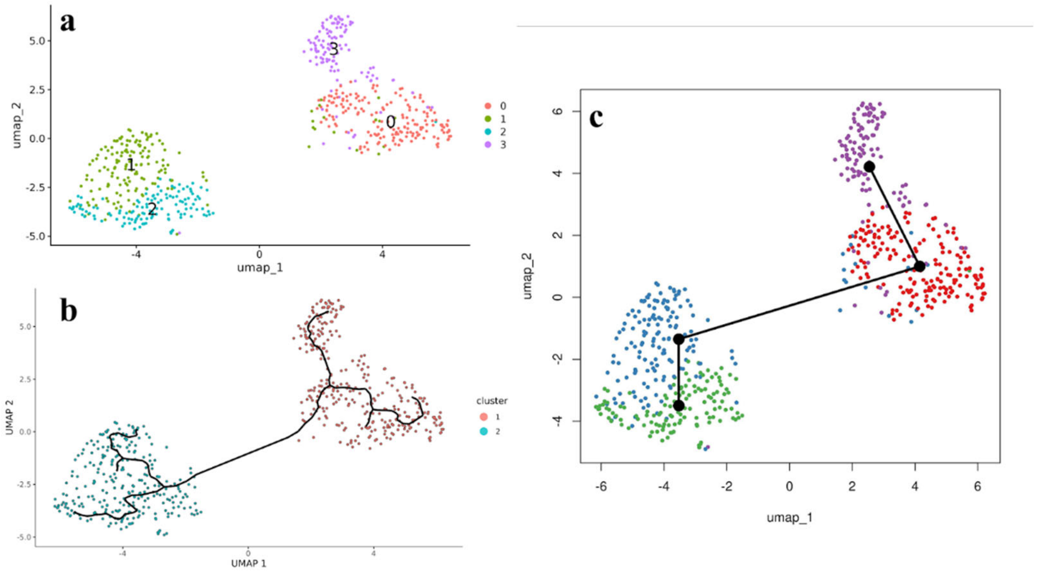

Figure S3. **Gbl13 (5 passage)** Glioblastoma sample analysis. (**a**) Clusters (**CellMarker DB** 0 - SLC16A7+ cells, 1 - FOXN4+ cells, 2 - Monocytes, 3 - Multilymphoid progenitor cells. **singleCellBase** 0 - Mesenchymal-like cells, 1 - Progenitor cells, 2 - Stem cells, 3 - Monocytes); (**b**) Monocle trajectory on clusters; (**c**) Slingshot trajectory

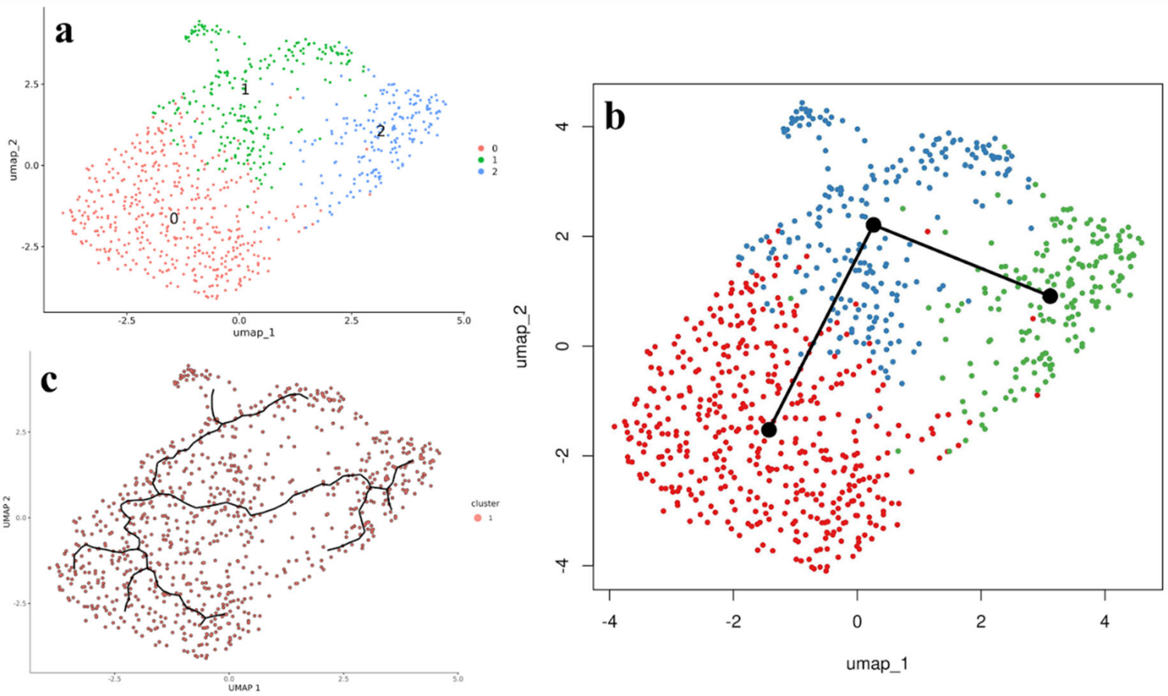

Figure S4. **Gbl27 (11 passage)** Glioblastoma sample analysis. (**a**) Clusters (**CellMarker DB** 0 - Monocytes, 1 - Neural progenitor cells, 2 - Neural progenitor cells. **singleCellBase** 0 - Fibroblasts, 1 - G2m_s npcs, 2 - Cell cycling cells); (**b**) Slingshot trajectory; (**c**) Monocle trajectory on clusters

Table S4. Cell types and biological process

Sample	Cluster	GO (Biological process)	Common markers (CellMarker DB, singleCellBase)
Gbl17	0 (DCLK1+ progenitor cells, Mesenchymal-like cells)	GO:0032963 Collagen metabolic process<br>GO:0001568 Blood vessel development	COL1A2, NGFR
	1 (Astrocytes, Fibroblasts-like cells)	no	CPE, SRPX, BEX1
	2 (Astrocytes, Erythrocytes)	GO:0006695 Cholesterol biosynthetic process<br>GO:0010273 Detoxification of copper ion	MT1E, MT1X, SLC3A2
	3 (Monocytes, G2m_s npcs)	GO:0097435 Supramolecular fiber organization<br>GO:0006265 DNA topological change<br>GO:0051256 Mitotic spindle midzone assembly<br>GO:0090267 Positive regulation of mitotic cell cycle spindle assembly checkpoint	no
	4 (Monocytes, Glia cells)	GO:0006098 Pentose-phosphate shunt<br>GO:0010917 Negative regulation of mitochondrial membrane potential	no

		GO:0035794 Positive regulation of mitochondrial membrane permeability GO:0006119 Oxidative phosphorylation GO:0006091 Generation of precursor metabolites and energy GO:0000028 Ribosomal small subunit assembly GO:0002181 Cytoplasmic translation GO:0042255 Ribosome assembly GO:0042274 Ribosomal small subunit biogenesis	
	5 (Astrocytes, Monocytes)	no	no
Gbl13	0 (SLC16A7+ cells, Mesenchymal-like cells)	GO:0030199 Collagen fibril organization	no
	1 (FOXN4+ cells, Progenitor cells)	no	UBE2T, CDT1
	2 (Monocytes, Stem cells)	HSA-2262752 Cellular responses to stress (RPL22L1, RPL8, ASNS, H2AZ1)	no
	3 (Multilymphoid progenitor cells, Monocytes)	no	no
Gbl27	0 (Monocytes, Fibroblasts)	no	no
	1 (Neural progenitor cells, G2m_s npcs)	GO:0051987 Positive regulation of attachment of spindle microtubules to kinetochore GO:0051256 Mitotic spindle midzone assembly GO:0051383 Kinetochore organization GO:0051256 Mitotic spindle midzone assembly	ASPM, BIRC5, CKAP2, CKS2, PBK, PRC1, PTTG1, SMC4, TOP2A, TPX2
	2 (Neural progenitor cell, Cell cycling cells)	GO:1905463 Negative regulation of DNA duplex unwinding GO:1902975 Mitotic DNA replication initiation GO:0000727 Double-strand break repair via break-induced replication GO:1902969 Mitotic DNA replication GO:0051095 Regulation of helicase activity	ATAD2, FEN1, HIST1H4C, KIF23, MCM2, MCM6, RRM1

Cell cultures that do not genetically correspond to the original tumor clone and exhibit a completely different set of mutations

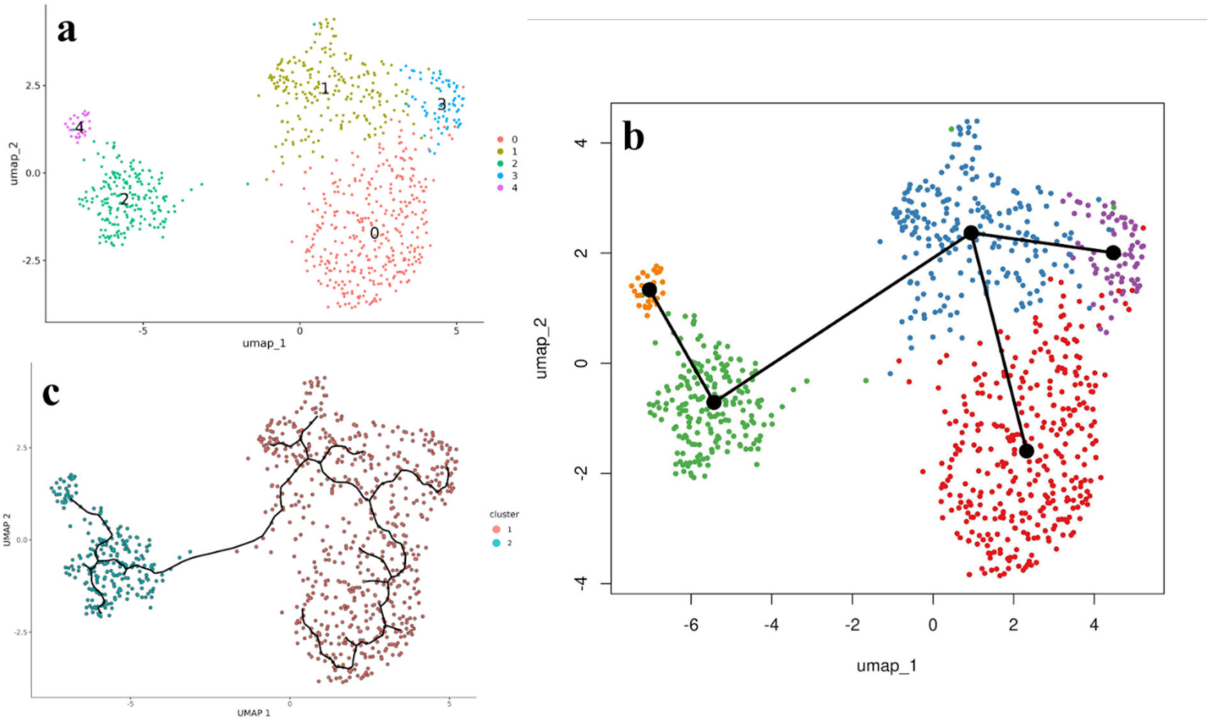

Figure S5. **Gbl24 (7 passage)** Glioblastoma sample analysis. (**a**) Clusters (**CellMarker DB** 0 - Neural progenitor cells, 1 - Monocytes, 2 - Monocytes, 3 - SLC16A7+ cells, 4 - Monocytes. **singleCellBase** 0 - Cell cycling cells, 1 - Fibroblasts, 2 - Glia cells, 3 - Endothelial cells, 4 - Dendritic cells); (**b**) Slingshot trajectory; (**c**) Monocle trajectory on clusters

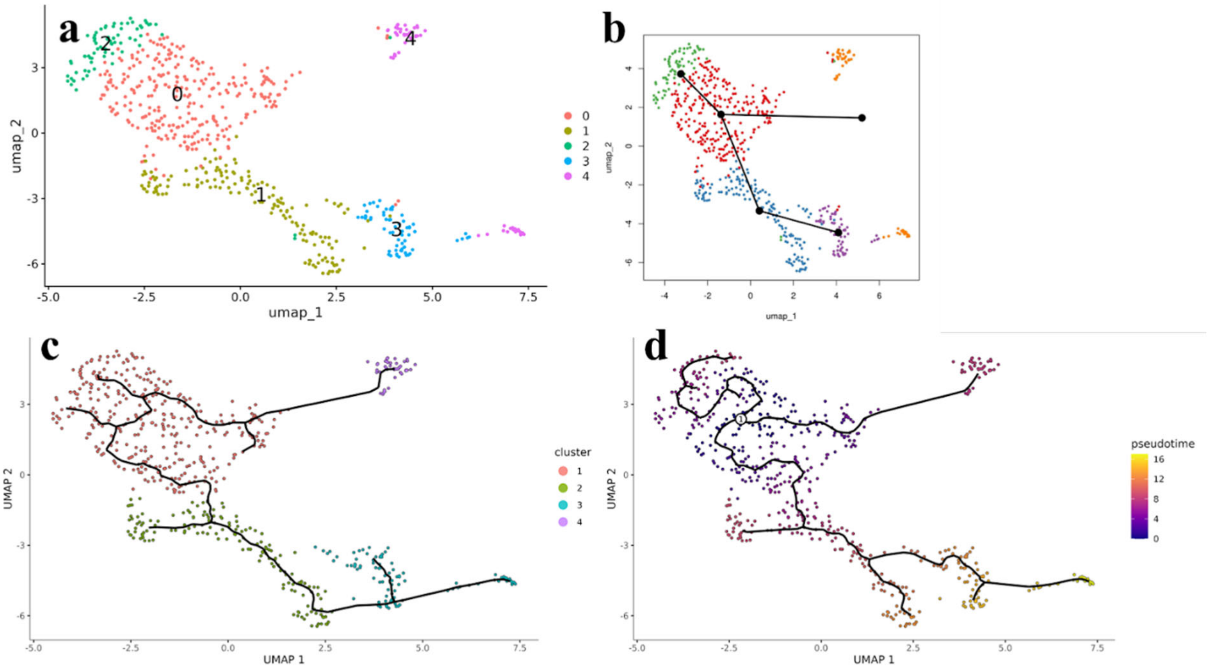

Figure S6. **Gbl6 (9 passage)** Glioblastoma sample analysis. (**a**) Clusters (**CellMarker DB** 0 - Astrocytes, 1 - Monocytes, 2 - Monocytes, 3 - Neural progenitor cells, 4 - SLC16A7+ cells. **singleCellBase** 0 - Neural cells, 1 - Pericytes, 2 - Endothelial cells, 3 - Cell cycling cells, 4 - Neural

cells); (**b**) Slingshot trajectory; (**c**) Monocle trajectory on clusters; (**d**) Monocle trajectory on pseudotime.

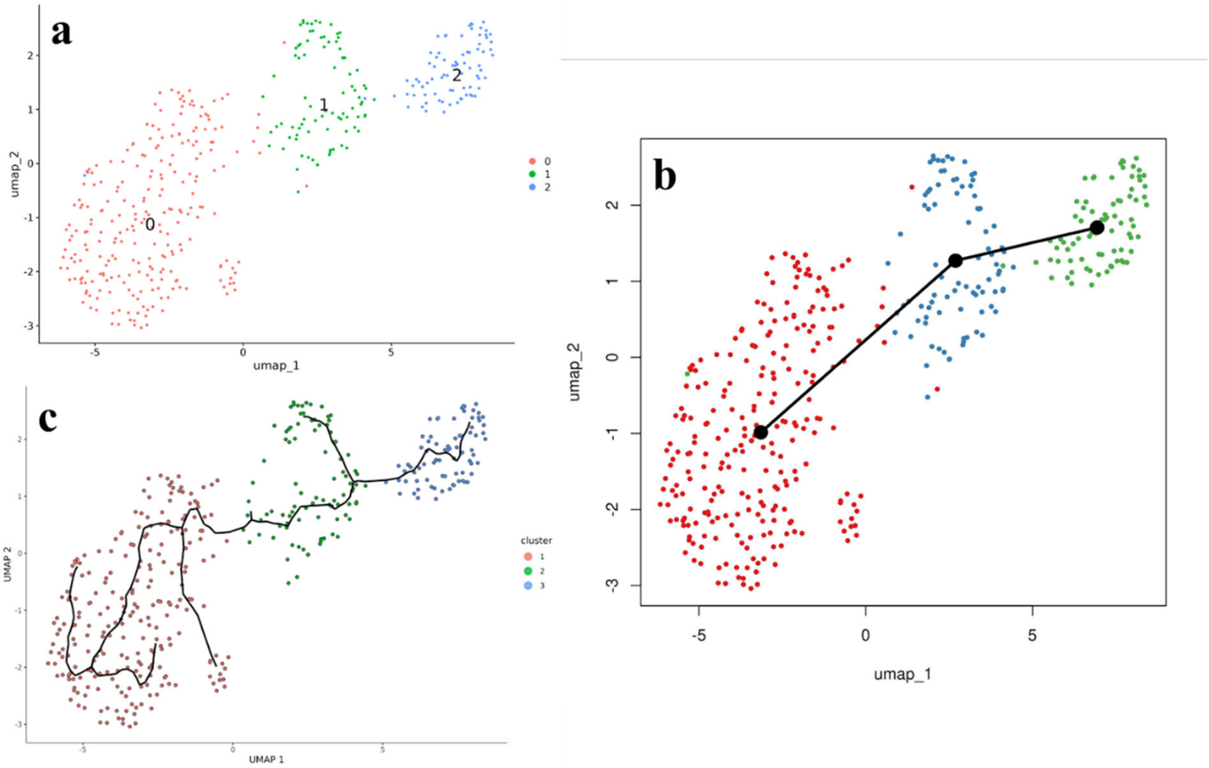

Figure S7. **Gbl28 (5 passage)** Glioblastoma sample analysis. (**a**) Clusters (**CellMarker DB** 0 - Microglial cells, 1 - Neural progenitor cells, 2 - Neural progenitor cells. **singleCellBase** 0 - Endothelial cells, 1 - Cell cycling cells, 2 - G2m_s npcs); (**b**) Slingshot trajectory; (**c**) Monocle trajectory on clusters

Table S5. Cell types and biological process

Sample	Cluster	GO (Biological process)	Common markers (CellMarker DB, singleCellBase)
Gbl24	0 (Neural progenitor cell, Cell cycling cells)	GO:0008315 G2/MI transition of meiotic cell cycle<br>GO:0032954 Regulation of cytokinetic process	no
	1 (Monocytes, Fibroblasts)	GO:0048333 Mesodermal cell differentiation<br>GO:0001704 Formation of primary germ layer<br>GO:0007498 Mesoderm development<br>GO:0050727 Regulation of inflammatory response<br>GO:0032496 Response to lipopolysaccharide<br>GO:0000307 Cyclin-dependent protein kinase holoenzyme complex	CDKN1A, CCNL1, CSRNP1
	2 (Monocytes, Glia cells)	GO:0006123 Mitochondrial electron transport,	no

		cytochrome c to oxygen<br>GO:0019646 Aerobic electron transport chain<br>GO:0042775 Mitochondrial ATP synthesis coupled electron transport<br>GO:0006119 Oxidative phosphorylation<br>GO:0006412 Translation	
	3 (SLC16A7+ cells, Endothelial cells)	GO:0043170 Macromolecule metabolic process<br>GO:0006807 Nitrogen compound metabolic process<br>GO:0048842 Positive regulation of axon extension involved in axon guidance<br>GO:0060385 Axonogenesis involved in innervation<br>GO:0032489 Regulation of Cdc42 protein signal transduction<br>GO:0072577 Endothelial cell apoptotic process<br>GO:0001954 Positive regulation of cell-matrix adhesion	ABI2, BMPR2
	4 (Monocytes, Glia cells)	GO:0006123 Mitochondrial electron transport, cytochrome c to oxygen<br>GO:0019646 Aerobic electron transport chain<br>GO:0042775 Mitochondrial ATP synthesis coupled electron transport<br>GO:0002181 Cytoplasmic translation<br>GO:0006518 Peptide metabolic process<br>GO:0022904 Respiratory electron transport chain	LGALS1, SNX3, UBA52
Gbl6	0 (Astrocytes, Neural cells)	GO:0006695 Cholesterol biosynthetic process	ALPL, GRIA1
	1 (Monocytes, Pericytes)	GO:0050839 Cell adhesion molecule binding	no
	2 (Monocytes, Endothelial cells)	HSA-168256 Immune System<br>GO:0060839 Endothelial cell fate commitment<br>GO:0032489 Regulation of Cdc42 protein signal transduction<br>GO:0010594 Regulation of endothelial cell migration	no

		GO:0010632 Regulation of epithelial cell migration<br>GO:0045765 Regulation of angiogenesis	
	3 (Neural progenitor cells, Cell cycling cells)	GO:0008315 G2/MI transition of meiotic cell cycle<br>GO:0032954 Regulation of cytokinetic process	
	4 (SLC16A7+ cells, Neural cells)	no	AKAP9
Gbl28	0 (Microglial cell, Endothelial cells)	GO:0106016 Positive regulation of inflammatory response to wounding<br>GO:0035455 Response to interferon-alpha<br>GO:0140467 Integrated stress response signaling	no
	1 (Neural progenitor cell, Cell cycling cells)	GO:0008315 G2/MI transition of meiotic cell cycle<br>GO:0032954 Regulation of cytokinetic process<br>GO:1902975 Mitotic DNA replication initiation	no
	2 (Neural progenitor cell, G2m_s npcs)	GO:0008315 G2/MI transition of meiotic cell cycle<br>GO:0051988 Regulation of attachment of spindle microtubules to kinetochore	CKAP2, CKS2, KNSTRN, NUF2, PBK, TOP2A
