# Supplementary figures and images for: Intratumoral Cell Heterogeneity in Patient-Derived Glioblastoma Cell Lines Revealed by Single-Cell RNA-Sequencing
Source: Int J Mol Sci. 2024 Aug 2;25(15):8472. doi: 10.3390/ijms25158472 (PMC11313325; doi:10.3390/ijms25158472)

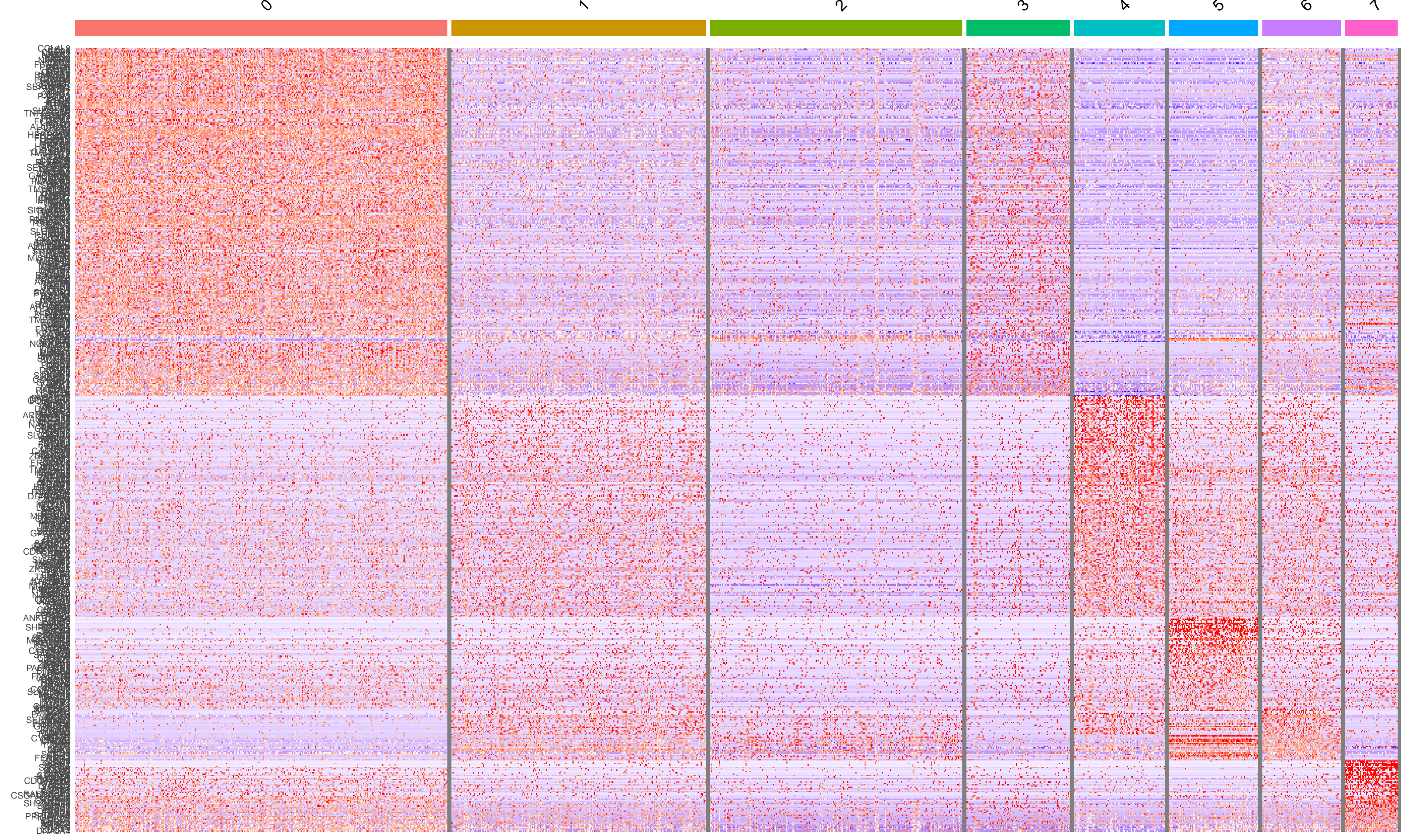

Supplement: Supplementary file 1 [file ijms-25-08472-s001.zip › Gb75t_heatmap.pdf]

0

1

2

3

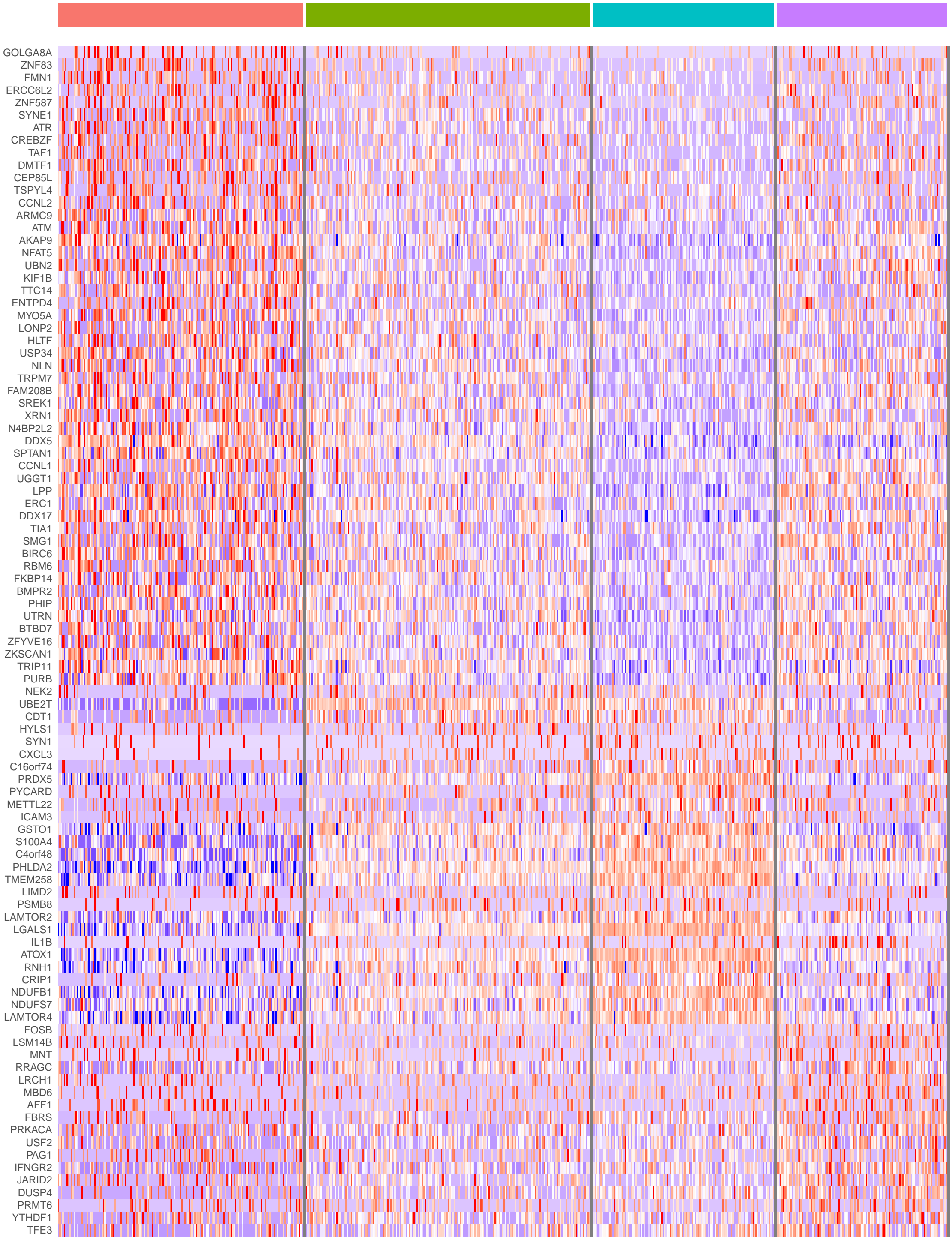

Supplement: Supplementary file 1 [file ijms-25-08472-s001.zip › Gbl13_heatmap.pdf]

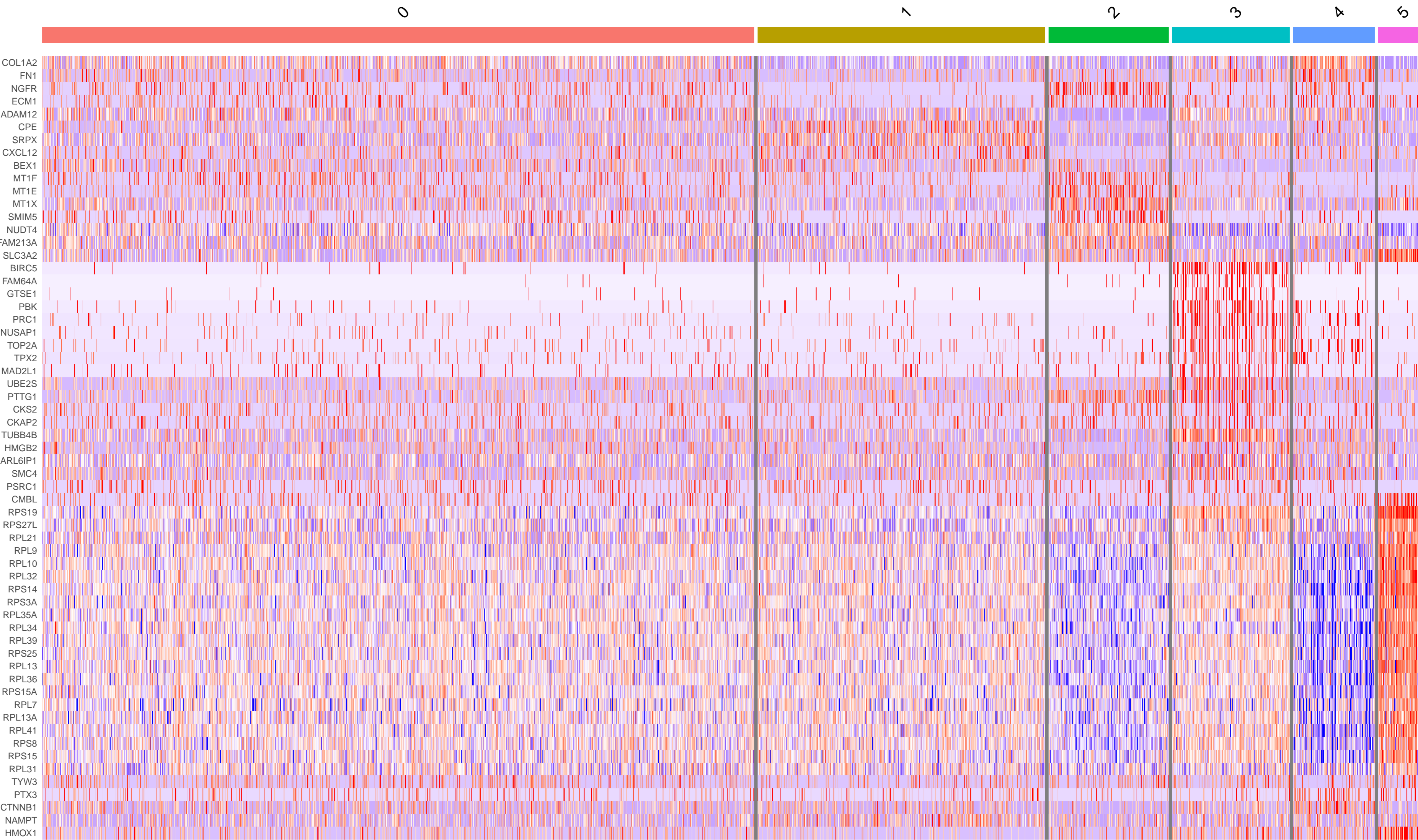

Supplement: Supplementary file 1 [file ijms-25-08472-s001.zip › Gbl17_heatmap.pdf]

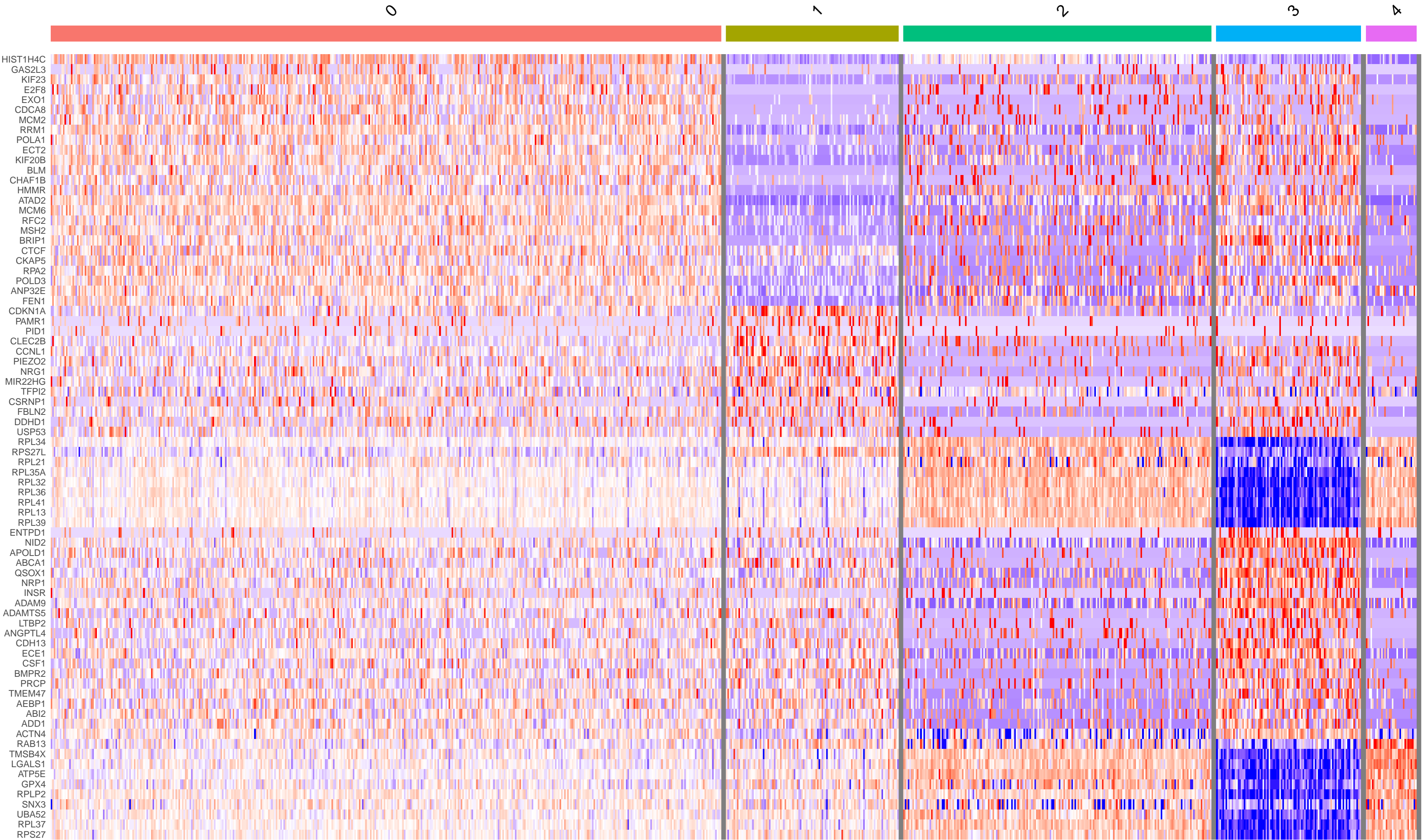

Supplement: Supplementary file 1 [file ijms-25-08472-s001.zip › Gbl24_heatmap.pdf]

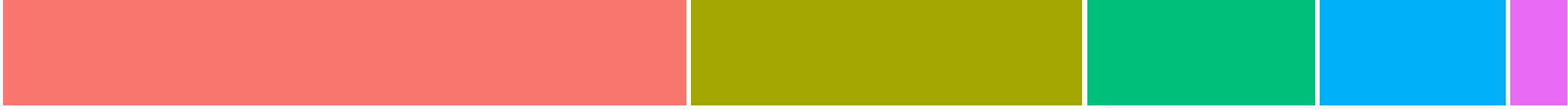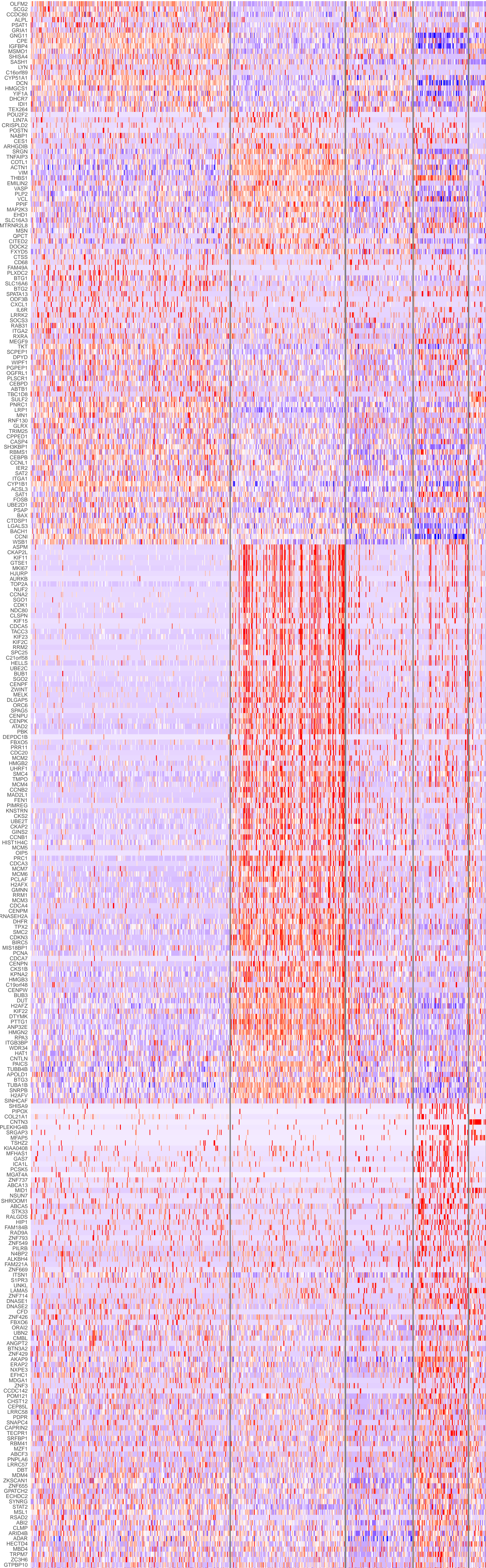

Supplement: Supplementary file 1 [file ijms-25-08472-s001.zip › Gbl6_heatmap.pdf]
